# A Strategy for O-Glycoproteomics of Enveloped Viruses—the O-Glycoproteome of Herpes Simplex Virus Type 1

**DOI:** 10.1371/journal.ppat.1004784

**Published:** 2015-04-01

**Authors:** Ieva Bagdonaite, Rickard Nordén, Hiren J. Joshi, Sally Dabelsteen, Kristina Nyström, Sergey Y. Vakhrushev, Sigvard Olofsson, Hans H. Wandall

**Affiliations:** 1 Copenhagen Center for Glycomics, Institute of Cellular and Molecular Medicine, University of Copenhagen, Copenhagen, Denmark; 2 Department of Clinical Virology, Sahlgrenska Academy, University of Gothenburg, Gothenburg, Sweden; 3 Institute of Odontology, University of Copenhagen, Copenhagen, Denmark; Institut Pasteur, FRANCE

## Abstract

Glycosylation of viral envelope proteins is important for infectivity and interaction with host immunity, however, our current knowledge of the functions of glycosylation is largely limited to N-glycosylation because it is difficult to predict and identify site-specific O-glycosylation. Here, we present a novel proteome-wide discovery strategy for O-glycosylation sites on viral envelope proteins using herpes simplex virus type 1 (HSV-1) as a model. We identified 74 O-linked glycosylation sites on 8 out of the 12 HSV-1 envelope proteins. Two of the identified glycosites found in glycoprotein B were previously implicated in virus attachment to immune cells. We show that HSV-1 infection distorts the secretory pathway and that infected cells accumulate glycoproteins with truncated O-glycans, nonetheless retaining the ability to elongate most of the surface glycans. With the use of precise gene editing, we further demonstrate that elongated O-glycans are essential for HSV-1 in human HaCaT keratinocytes, where HSV-1 produced markedly lower viral titers in HaCaT with abrogated O-glycans compared to the isogenic counterpart with normal O-glycans. The roles of O-linked glycosylation for viral entry, formation, secretion, and immune recognition are poorly understood, and the O-glycoproteomics strategy presented here now opens for unbiased discovery on all enveloped viruses.

## Author Summary

Information on site-specific O-glycosylation of viral envelope glycoproteins is generally very limited despite important functions. We present a powerful mass-spectrometry based strategy to globally identify O-glycosylation sites on viral envelope proteins of a given virus in the context of a productive infection. We successfully utilized the strategy to map O-linked glycosylation sites on the complex HSV-1 virus demonstrating that O-glycosylation is widely distributed on most envelope proteins. Moreover, we used genetically engineered keratinocytes lacking O-glycan elongation capacity to demonstrate that O-linked glycans are indeed important for HSV-1 biology as HSV-1 particles produced in these cells had significantly lower titers compared to wild-type keratinocytes. These tools enable wider discovery and detailed analysis of the role of site-specific O-glycosylation in virology.

## Introduction

Enveloped viruses contain one or more membrane proteins important for adhesion and entry to host cells [1]. The majority of envelope membrane proteins are predicted or confirmed to be covered with glycans with important functions in protein folding, transport, formation of infectious particles, entry into host cells, and shielding from the host’s immune system [2–7]. Numerous studies have addressed the structures and functions of N-linked glycans on membrane glycoproteins from different viruses [8–13], and N-glycosylation has attracted particular attention for the human immunodeficiency virus (HIV), where a cluster of N-glycans constitute the epitope for the 2G12 and other antibodies with broadly neutralizing function [14, 15]. In striking contrast, information on O-linked glycans and, in particular, where O-glycans are found is generally missing, which leaves a void in knowledge of the biological functions of O-glycosylation. This is in spite of substantial evidence suggesting that O-glycosylation is important for viral infectivity and virus-induced immunomodulation for several viruses [4, 7, 16–18].

Viral proteins destined for the virion surface travel through the host’s secretory pathway where they hijack the host cell’s glycosylation machinery and get decorated with glycans [19]. Protein glycosylation is controlled by hundreds of glycosyltransferases that reside in the secretory pathway and that, in a non-template fashion, orchestrate the diversity of glycan structures found on proteins [20]. There is substantial evidence that many viral membrane proteins are N-glycosylated, although there is surprisingly limited experimental evidence for actual glycosylation sites for many viruses with few exceptions [21, 22]. However, to a large extent the consensus sequence motif NXS/T (X—all amino acids except P) enables reliable prediction of N-glycosites [23]. There is less evidence for the presence of O-glycosylation (GalNAc-type) on virus membrane glycoproteins, and this largely relies on the presence of mucin-like sequence motifs with high density of PST residues. Such are found in e.g. HSV-1 gC [24] and Ebola virus glycoprotein [25], but recent studies suggest that O-glycosylation is more prevalent in non-mucin-like regions and often exist as isolated sites or in small clusters [26]. Site-specific O-glycosylation in such isolated or clustered positions may exert co-regulatory functions of basic processes such as pro-protein processing and ectodomain shedding [27], which may affect viral fusion protein activation and function [28, 29]. In contrast to N-linked glycosylation that can be predicted with reasonable certainty our knowledge of O-glycosylation is hampered by lack of simple consensus motifs for prediction of O-glycosites. O-glycosylation is unique in being controlled by 20 polypeptide GalNAc-transferases (GalNAc-Ts) that transfer GalNAc to select Ser, Thr and, possibly, Tyr residues [30]. The initial GalNAc residues are further elongated, branched, and capped by a large number of different glycosyltransferases in subsequent processing steps. The large number of GalNAc-T isoenzymes with distinct peptide substrate specificities and cell expression patterns provides a high degree of differential regulation of O-glycosylation capacity directed by the repertoire of GalNAc-Ts in a given cell. This unprecedented complexity of protein glycosylation adds to the need for direct experimental analysis of O-glycosylation in the appropriate cellular context to probe biological functions. It is therefore essential to develop strategies to enable characterization of the O-glycoproteomes of viruses produced in representative host cells during virus infection.

**Fig 1:**
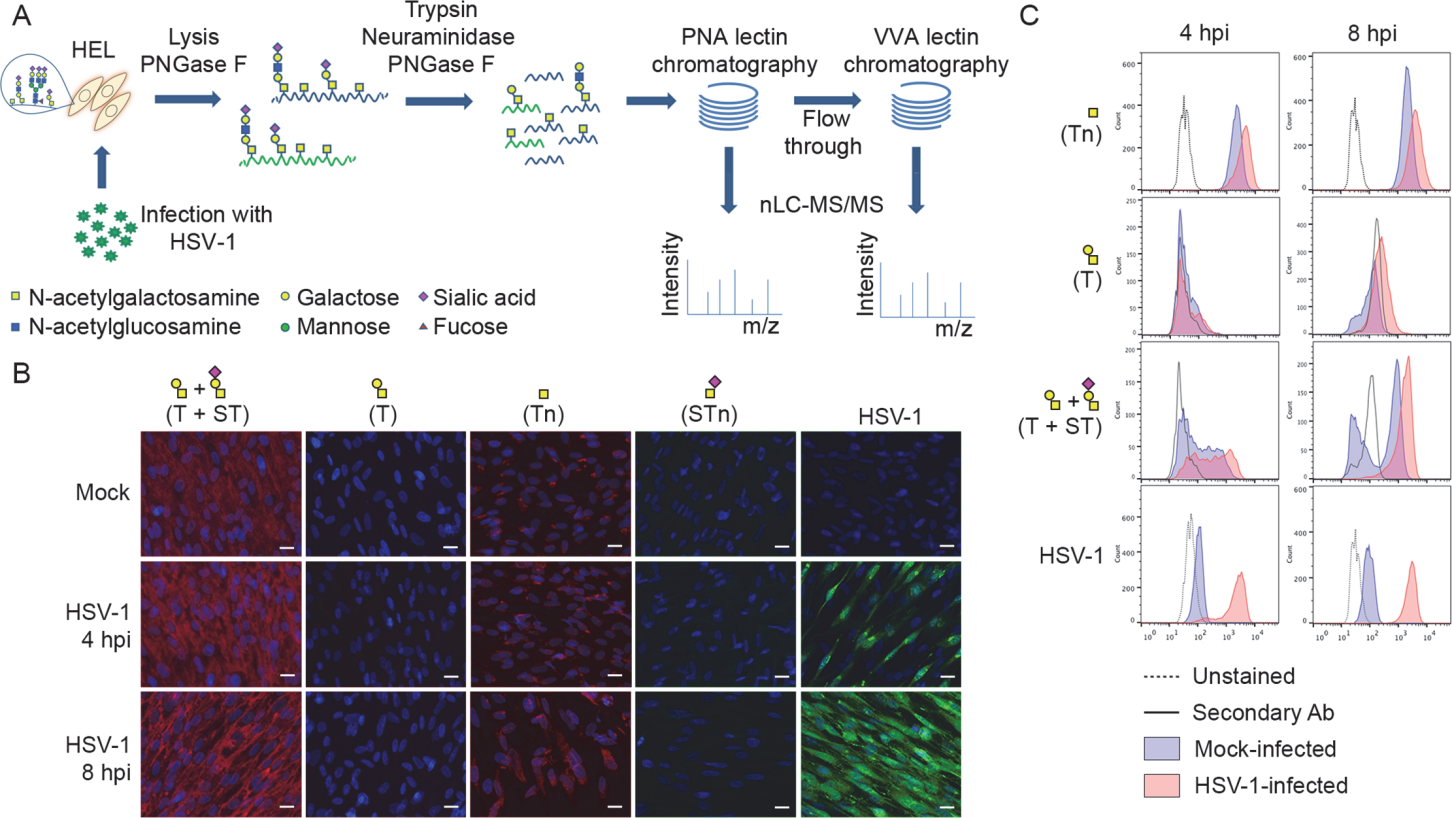
Glycopeptide enrichment strategy and glycoprofiling of human embryonic lung (HEL) fibroblasts. (A) A schematic representation of protein digestion and glycopeptide enrichment strategy for glycoproteomic analysis. (B) Glycoprofiling of mock- or HSV-1 Syn17+ infected (MOI of 10) HEL fibroblasts fixed and permeabilized at indicated time points. A panel of carbohydrate-specific monoclonal antibodies was used for immunofluorescent staining: 3C9 mAb (T structure; Galβ1-3GalNAc1α-O-Ser/Thr); 5F4 mAb (Tn structure; GalNAcα1-O-Ser/Thr); 3F1 mAb (STn structure; Neu5Acα2-6GalNAcα1-O-Ser/Thr). ST structure (Neu5Acα2-3Galβ1-3GalNAcα1-O-Ser/Thr) was detected using 3C9 mAb plus neuraminidase treatment. HSV-1 was detected using a FITC-conjugated polyclonal Ab. Hpi—hours post-infection; scale bar—20 μm. (C) Carbohydrate profile of permeabilized HEL fibroblasts analyzed by flow cytometry at indicated time points. Tn structure (GalNAcα1-O-Ser/Thr) was detected using FITC-conjugated *Helix pomatia* agglutinin (HPA), other labels as in Fig 1B. HSV-1 infected samples were gated (S1 Fig) on HSV-1-positive cells (except for HPA-FITC labeled samples).

To address this need, we used herpes simplex virus type 1 (HSV-1) as a model to develop a comprehensive viral O-glycoproteomics strategy. We first determined the major O-glycan structures produced during virus infection, and used this to design a two-step sequential lectin enrichment strategy for capture of desialylated O-glycopeptides in total proteolytic digests of infected cells (Fig 1A). The strategy is based on our recent “SimpleCell” approach for O-glycoproteomics [26, 31], but extended to enable sensitive mapping of O-glycosites in cells with the common sialylated core 1 O-glycosylation capacity such as found in human embryonic lung (HEL) fibroblasts. Applied to HSV-1 as a proof-of-concept, we provide the first comprehensive HSV-1 O-glycoproteome with identification of 8 of the 12 HSV-1 envelope proteins as O-glycoproteins with a total of 74 unique O-glycosites. We further took advantage of an isogenic cell model in the human keratinocyte (HaCaT) cell line in which productive HSV-1 infection can be established, and provide evidence that O-glycan elongation has functional consequences for virus production and infectivity. The strategies and findings presented may have important bearings for vaccine design.

## Results

### Glycophenotyping of HEL fibroblasts

We glycoprofiled mock or HSV-1 infected HEL fibroblasts using a panel of well characterized monoclonal antibodies to core 1 O-glycans. HEL fibroblasts infected with HSV-1 predominantly expressed sialylated core 1 O-glycan structure ST (Neu5Acα2-3Galβ1-3GalNAcα1-O-Ser/Thr) and truncated O-glycan structure Tn (GalNAcα1-O-Ser/Thr) (Fig 1B and 1C). In order to have a more comprehensive view of O-glycan repertoire we also performed chemical glycan release by reductive β-elimination and analyzed the native glycans by direct infusion nanoESI-MS. Glycomic analysis in negative polarity (S4A and S4C Fig) identified the majority of O-glycans as mono- or disialylated T structures in mock- and HSV-1-infected fibroblasts. Due to a potential tendency of sialylated glycans to be ionized better at the negative polarity compared to neutral ones, we have analyzed the same samples at the positive ion mode as well. As it is shown in S4B and S4D Fig, sialylated core 1 O-glycan structures represented the most abundant class of O-glycans in both mock- and HSV-1 infected fibroblasts. Non-sialylated T structures (Galβ1-3GalNAc1α-O-Ser/Thr) and various core 2 O-glycan structures (non-, mono-, and disialylated) were also present at lower levels. In conclusion, mock- and HSV-1-infected fibroblasts exhibited similar O-glycan profiles with predominantly sialylated core 1 O-glycan structures.

### Identification of O-glycosites on HSV-1 envelope glycoproteins

Given the finding that infected HEL cells expressed both ST/T and Tn glycans, we developed a two-step lectin enrichment strategy to enable identification of O-glycosites. Enrichment of glycopeptides in total protease digest of complex mixtures of proteins is essential for sensitive detection. The strategy to identify O-glycosylation sites in HSV-1 is depicted in Fig 1A. Cell lysates from HSV-1 infected cells and released virions were digested sequentially with trypsin and neuraminidase. We first employed peanut agglutinin (PNA) Lectin Weak Affinity Chromatography (LWAC) to capture T-glycopeptides and the flow through of this step was further subjected to *Vicia villosa* lectin (VVA) LWAC to capture Tn-glycopeptides [32] (Fig 1A). The elution fractions of PNA and VVA LWACs were analyzed by tandem mass spectrometry equipped with ETD fragmentation to identify O-linked glycosylation sites on HSV-1 envelope glycoproteins. By using this strategy we identified eight out of the 12 HSV-1 envelope glycoproteins and a total of 74 unique O-glycosylation sites. Nearly all of these O-glycosites (72 sites) were identified in total lysates of infected cells, while direct analysis of released virions resulted in identification of 20 O-glycosites of which only two were not found in the lysate. Comparing identifications from PNA and VVA LWACs, fewer glycosites (43 sites) were identified from PNA LWAC than from VVA (58 sites) (Tables 1 and S1). However, at least one third of the sites identified with PNA LWAC were non-redundant with VVA LWAC identified sites. The direct analysis of virions yielded markedly lower number of O-glycosylation sites (10 sites with each VVA and PNA LWAC) (Table 1).

**Table 1:** Overview of site identification using different enrichment strategies.

	PNA and VVA		PNA			VVA		
	Totalsites	Unambiguoussites	Totalsites	Unambiguoussites	Viral proteins	Totalsites	Unambiguoussites	Viral proteins
Viral particles	20	4	10	4	gD, gE, gG	10	0	gB, gC, gL
Cell lysate	72	60	41	28	gB, gC, gD, gE, gG, gH, gI, gL	58	53	gB, gC, gD, gE, gG, gI, gL
Total	74	62	43	30	gB, gC, gD, gE, gG, gH, gI, gL	58	53	gB, gC, gD, gE, gG, gI, gL

**Fig 2:**
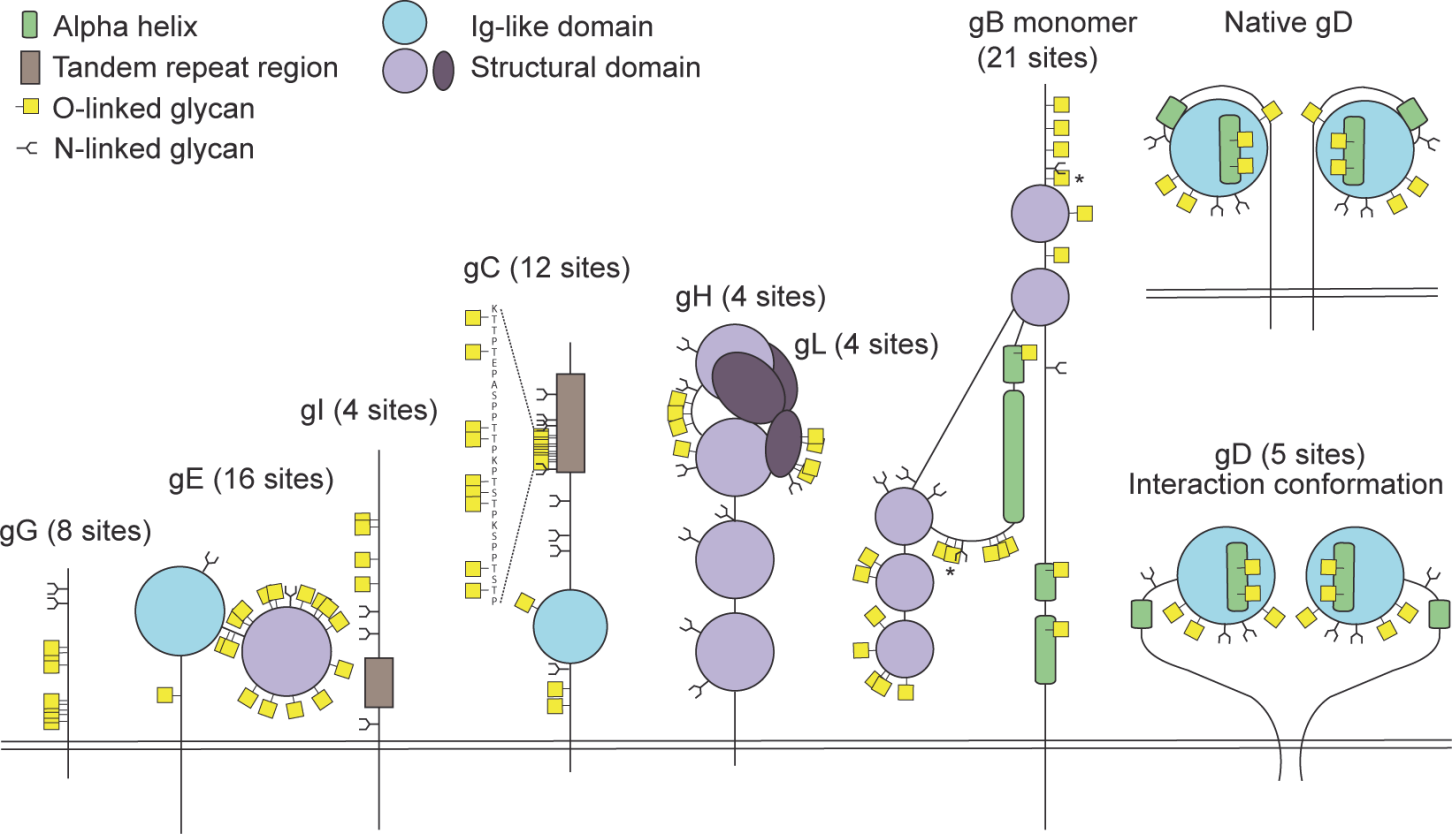
Identified O-linked glycosylation sites on HSV-1 envelope glycoproteins. The cartoon depicts approximate localization of the 74 identified O-linked glycosylation sites in the context of known structural elements of 8 HSV-1 envelope glycoproteins [38–42, 48]. The remaining 4 HSV-1 envelope glycoproteins without identified O-glycosylation are not depicted, although some of them are predicted to be N-glycosylated (gJ, gK, gM, gN). O-glycosylation sites marked with an asterisk can potentially have a slightly different location due to the ambiguity of the site identification within the peptide stretch. Sequence-predicted N-linked glycosylation sites are indicated.

**Fig 3:**
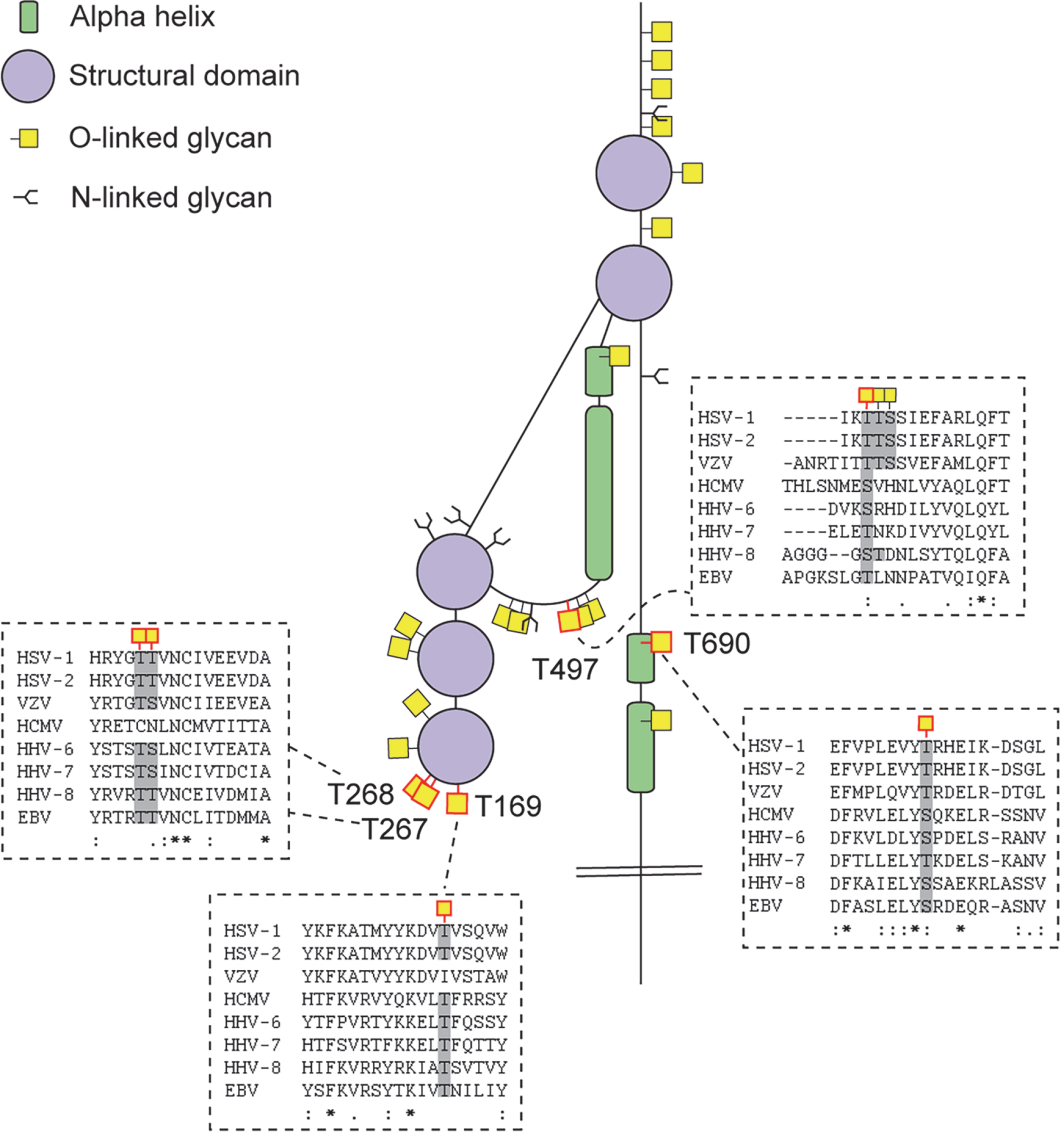
Conservation of O-linked glycosylation sites within the ectodomain of glycoprotein B between human herpesviruses. ClustalW2 multiple sequence alignment program was used to align amino acid sequences of glycoprotein B ectodomain between the reference strains of members of the *Herpesviridae* family. Structural depiction of glycoprotein B is shown. HSV-1 gB glycosylation sites at conserved serines/threonines between the aligned sequences are indicated with red outlined O-linked glycan icons. Dashed boxes show the multiple sequence alignment output for the sequences flanking the highly conserved glycosylated amino acids (marked with grey) between the *Herpesviridae* family members. Two ambiguous O-glycosylation sites within peptide stretch 265-YGTT-268 were allocated to canonical O-GalNAc acceptor amino acids (T267 and T268). HSV-1—human Herpes simplex virus type 1 (strain 17), HSV-2—human Herpes simplex virus type 2 (strain HG52), VZV—Varicella-zoster virus (strain Dumas), HCMV—human cytomegalovirus (strain Merlin), HHV-6—human herpesvirus 6A (strain Uganda-1102), HHV-7—human herpesvirus 7 (strain JI), HHV-8—Kaposi’s sarcoma-associated herpesvirus (isolate GK18), EBV—Epstein-Barr virus (strain AG876).

Fig 2 presents a graphic depiction of the HSV-1 O-glycoproteome. A total of 34 out of 74 identified O-glycosylation sites were localized on the four HSV-1 membrane proteins, gB, gD, gH and gL, which are all essential for viral infectivity *in vitro* [33–36] (Table 2). Twenty-one glycosylation sites were identified in gB, which is essential for fusion with host cell membrane [33]. The identified glycosylation sites include two positions, T53 and T480 (S1 Table), which have previously been proposed to be important for the interaction with the paired immunoglobulin-like type 2 receptor α based on the finding that Ala substitutions resulted in loss of interaction [37]. In addition, O-linked glycans were found throughout the ectodomain and localized to both ordered and unstructured regions of the molecule [38] (Fig 2). Interestingly, several gB O-glycosylation sites were highly conserved between 8 members of the human herpesviruses (Figs 3 and S2).

**Table 2:** Number of sites identified on individual envelope glycoproteins.

Envelope glycoprotein	Function	Total sites	Unambiguous sites	Ambiguous sites	PNA	VVA
gB	Attachment/fusion [33, 75]	21	17	4	9	19
gC	Attachment, complement receptor [43, 44]	12	11	1	1	12
gD	Interaction with entry receptors [71–73]	5	4	1	5	4
gE	Spread, F_c_ receptor [46, 47]	16	14	2	14	14
gG	Chemokine binding [51]	8	8	0	7	2
gH	Entry [35, 76]	4	0	4	4	0
gI	Spread, F_c_ receptor [46, 47]	4	4	0	1	3
gL	Entry [36, 76]	4	4	0	2	4

Glycoprotein D, which is necessary for virus entry into host cells [34], was glycosylated at five sites (Fig 2). Two of the identified O-glycosites, S93 and S100, were located within the Ig-like core of the molecule. Interestingly, two more glycans (T255 and Y259/S260) were situated on the Ig-core-flanking functional alpha-helix important for maintaining the unliganded conformation of the molecule as well as interaction with nectin-1 [39–41]. One additional site, S33, was located within the N-terminal motile region involved in the interaction with the entry receptor HVEM [39]. In glycoprotein H, which is also required for HSV-1 entry into permissive cells [35], we found four ambiguous O-glycosylation sites at the N-terminus (Fig 2). Three of the four sites were situated within a disordered region between two structural subdomains [42]. The peripheral membrane protein gL, that forms a heterodimer with gH, carried four O-glycans all of which were located within a poorly structured region of the molecule [42] (Fig 2). No sites were found within the protein-protein interaction regions of the two proteins.

The remaining 40 O-glycosites were distributed among four HSV-1 glycoproteins (gC, gE, gI and gG), which are all important for virus-host interaction and modulation of the host immune response (Table 2, Fig 2). Glycoprotein C is involved in initial attachment to heparan sulphate proteoglycans as well as immune evasion by acting as a complement receptor [43, 44], and is known to contain a glycosylated mucin-like tandem repeat region [45]. Accordingly, 9 of the identified sites were localized within the mucin-like region, while 3 sites were found outside of the tandem repeat region (Fig 2). Glycoprotein E forms an Fc receptor together with glycoprotein I and is known to facilitate cell-to-cell spread [46, 47]. Interestingly, we identified 16 O-glycosylation sites, of which many densely covered the N-terminal domain of the molecule, whereas the Fc-binding domain did not carry any O-glycans [48] (Fig 2). Four O-glycosylation sites were identified on glycoprotein I (Fig 2), one of which was situated within the region required for the Fc receptor function [49]. Unfortunately, we were not able to identify any of the sites, which are known to be glycosylated within the tandem repeat region of gI [50]. It is known that glycans within this mucin-like region of gI are much more closely spaced as compared to gC, thus it is very likely that trypsin digestion is inefficient within the very tightly glycosylated region of gI. Finally, we detected eight O-glycosylation sites on the chemokine-binding [51] glycoprotein G (Fig 2).

### HSV-1-induced Golgi fragments retain the micro-organization of intact Golgi apparatus

**Fig 4:**
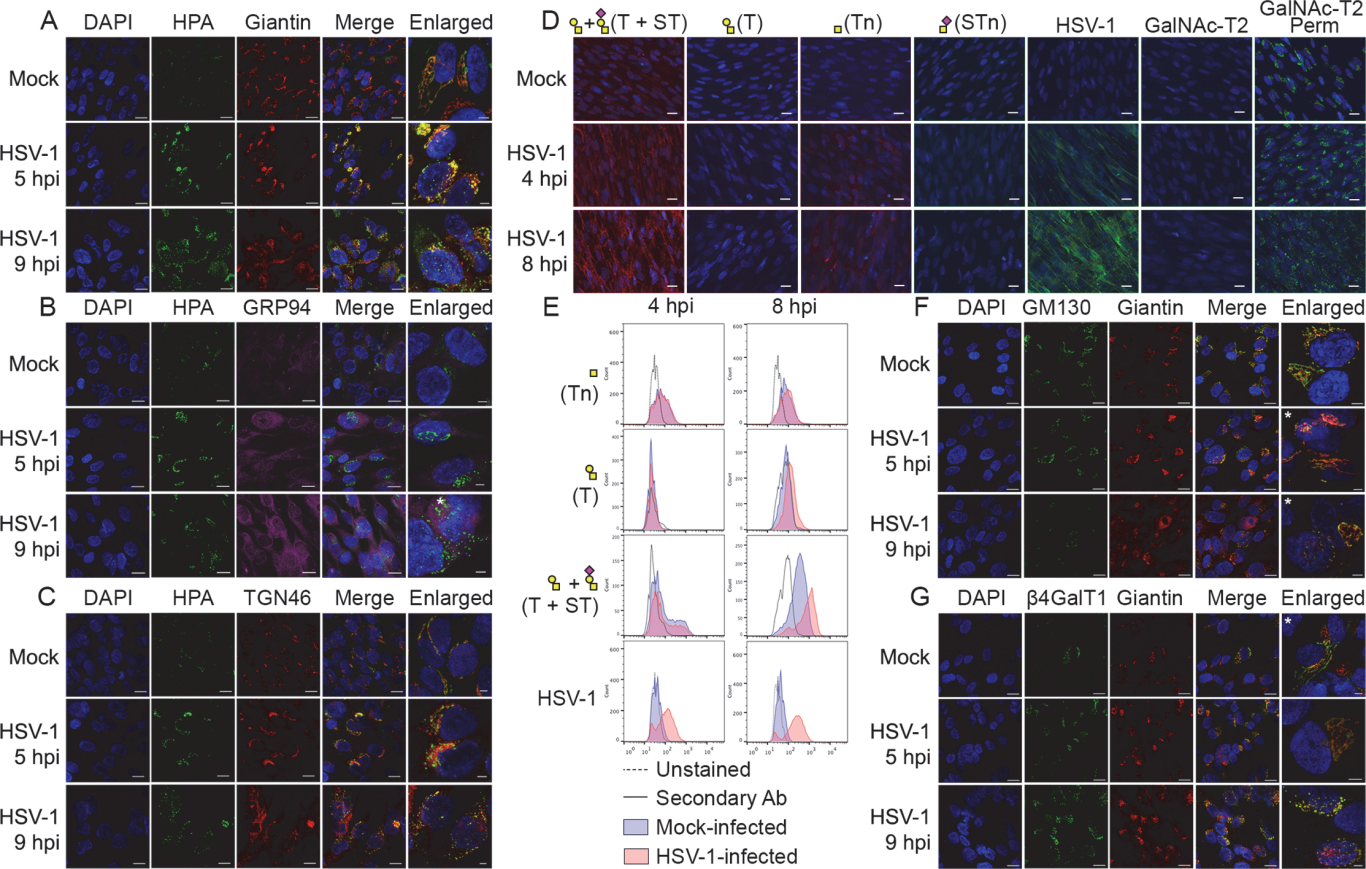
O-glycan processing upon HSV-1-induced fragmentation. (A-C) Immunolabeling of HSV-1 Syn17+ infected (MOI of 10) HEL fibroblasts fixed and permeabilized at indicated time points (hpi—hours post infection). Mock-infected cells were used as control. Cells were double labeled with antibodies and lectins and analyzed by confocal microscopy in order to investigate the cellular localization of Tn structures upon HSV-1 infection. (A) Green—HPA (Tn structure (GalNAcα1-O-Ser/Thr)); red—giantin (cis-/medial-Golgi marker); blue—DAPI. Scale bars: 20 μm for lower magnification images and 5 μm for higher magnification images. (B) Green—HPA; purple—GRP94 (ER marker); blue—DAPI. Scale bars as in Fig 4A. (C) Green—HPA; red—TGN46 (trans-Golgi network marker); blue—DAPI. Scale bars as in Fig 4A. (D, E) Cell surface expression of common O-glycoforms. HEL fibroblasts were mock- or HSV-1 Syn17+ infected (MOI of 10) and harvested at indicated time points. (D) Immunofluorescent cell surface staining using a panel of carbohydrate specific antibodies (Tn, mAb 5F4; STn, mAb 3F1; T, mAb 3C9; ST, mAb 3C9 plus neuraminidase treatment). 4C4 mAb for Golgi-resident glycosyltransferase GalNAc-T2 was used as a control for cell membrane integrity. Permeabilized cells (Perm) were used as a positive control for GalNAc-T2 staining. Scale bar—20 μm. (E) Cell surface carbohydrate profile of HEL fibroblasts analyzed by flow cytometry (Tn, HPA-FITC; T, mAb 3C9; ST, mAb 3C9 plus neuraminidase treatment). HSV-1 infected samples were gated (S1 Fig) on HSV-1-positive cells (except for HPA-FITC labeled samples). (F-G) HEL fibroblasts were mock- or HSV-1 Syn17+ infected (MOI of 10) and then fixed and permeabilized at indicated time points (hpi—hours post infection). Cells were double labeled with antibodies and analyzed by confocal microscopy in order to investigate the Golgi microorganization upon HSV-1 infection. (F) Green—GM130 (cis-Golgi marker); red—giantin (cis-/medial-Golgi marker); blue—DAPI. Scale bars as in Fig 4A. (G) Green—β4GalT1 (trans-Golgi marker); red—giantin (cis-/medial-Golgi marker); blue—DAPI. Scale bars as in Fig 4A. Enlarged micrographs marked with an asterisk do not correspond to the merged images to the left.

HEL fibroblasts normally produce complete O-glycans with fully sialylated core 1 structures (Fig 1B and 1C), but as shown here, infection with HSV-1 resulted in marked intracellular expression of truncated O-glycans (Tn), which was confirmed by our two-step O-glycoproteomics strategy where a substantial number of truncated Tn-glycopeptides were identified in mixture with T-glycopeptides (Fig 1, Tables 1 and S1). This prompted us to further investigate the effect of HSV-1 infection on O-glycan synthesis in more detail. Immunofluorescent staining for the truncated Tn O-glycan structure in permeabilized HSV-1-infected cells showed a Golgi-like staining pattern with numerous dispersed vesicle-like structures throughout the cytoplasm. The intracellular Tn expression increased along the course of infection with a complete dispersal of Tn staining throughout the cell after 9 hours (Fig 4A and 4C, HPA). Tn expression also partially co-localized with gC, implying that envelope glycoproteins are indeed O-glycosylated in the fragmented Golgi (S3 Fig). Despite the high expression of Tn inside the infected cells, we only detected small amounts of Tn on the surface as evaluated without permeabilization (Fig 4D and 4E). Both HSV-1 and mock-infected cells predominantly expressed elongated and sialylated O-glycans on the surface (Fig 4D and 4E). Co-localization studies showed that the intracellular Tn-positive structures in HSV-1-infected cells were highly correlated with the Golgi marker giantin during early stages of infection (Fig 4A: HSV-1 5 hpi), whereas lower degree of co-localization was observed late in infection (Fig 4A: HSV-1 9 hpi). There was no correlation between expression of Tn and the ER marker GRP94 in either mock- or HSV-1-infected cells (Fig 4B). However, partial co-localization was observed with trans-Golgi network marker TGN46 in heavily infected cells (Fig 4C: HSV-1 9 hpi).

To further evaluate how the infection impacted the organization of Golgi apparatus, we next investigated the relative localization of cis- and trans-Golgi markers during the course of HSV-1 infection. In HEL fibroblasts the classical Golgi markers GM130, giantin and β4Gal-T1 were redistributed into several distinct punctuate vesicular-like structures most likely representing remnants of Golgi structures as previously described [52]. Both cis-Golgi-resident GM130 and cis-/medial-Golgi-specific giantin were detected in close proximity within infection-induced Golgi fragments (Fig 4F: HSV-1 5 hpi, 9 hpi). Similarly, giantin and trans-Golgi-resident β4Gal-T1 were highly correlated within discrete Golgi fragments of infected cells (Fig 4G: HSV-1 5 hpi, 9 hpi). These findings suggest that the individual vesicular-like structures in HSV-1-infected HEL fibroblasts mirror the composition of an intact Golgi apparatus and potentially contain all the glycosyltransferases required for O-glycan synthesis and elongation.

### Elongated O-glycans are important for HSV-1 infectivity

**Fig 5:**
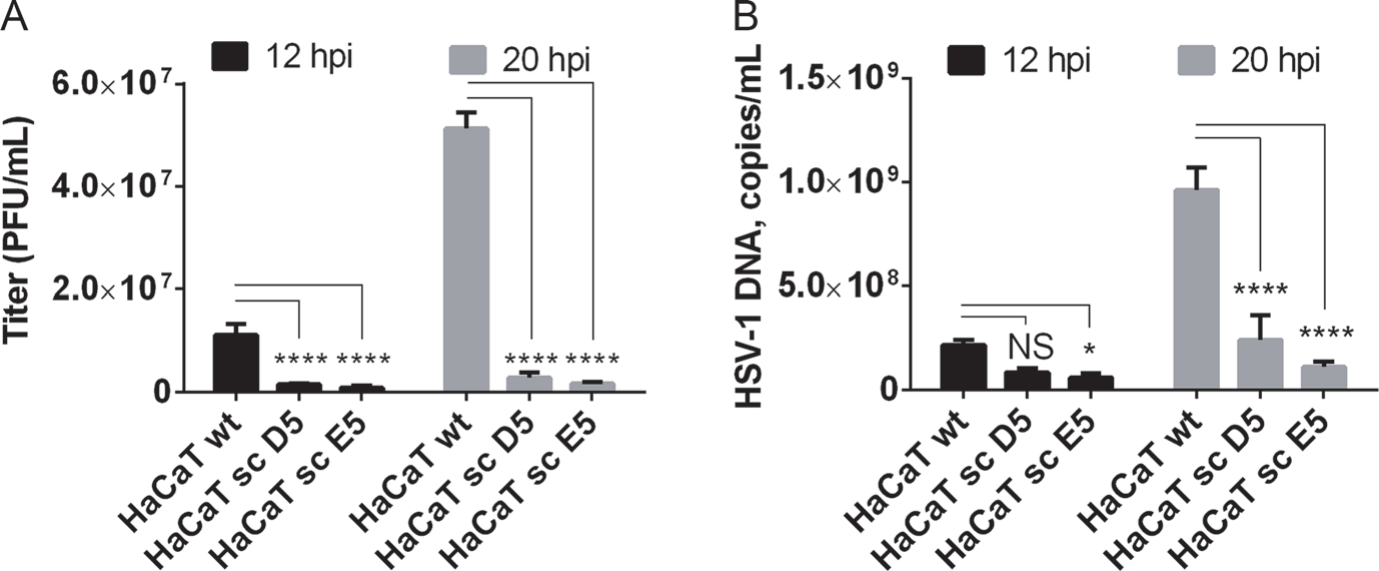
Elongation of O-linked glycans affects HSV-1 secretion/infectivity. (A) HaCaT wild-type or HaCaT mutant keratinocytes, lacking elongation of mucin-type O-liked glycosylation (HaCaT sc D5 and HaCaT sc E5) were infected with MOI of 10 of HSV-1 Syn17+ produced in HaCaT wt. Medium was harvested at 12 and 20 hours post-infection and number of infectious particles were quantified using plaque titration on Vero culture monolayer and expressed as plaque forming units per mL of medium (PFU/mL). Bar graphs represent mean values of 3 biological replicates assayed by 2 technical replicates each + SD. A 2-way ANOVA with Tukey’s multiple comparison test was used to compare differences between means. NS—*p* > 0.05, *—*p* < 0.05, **—*p* < 0.01, ***—*p* < 0.001, ****—*p* < 0.0001. Results are representative of at least two independent experiments. (B) Numbers of viral DNA copies in the medium were quantified by qPCR and using a standard curve based on amplification of known copy numbers of HSV-1 DNA fragments cloned in Topo TA plasmids. Copy numbers of viral DNA are expressed as copies/mL. Bar graphs represent mean values of 3 biological replicates assayed by 3 technical replicates each + SD. A 2-way ANOVA with Tukey’s multiple comparison test was used to compare differences between means. NS—*p* > 0.05, *—*p* < 0.05, **—*p* < 0.01, ***—*p* < 0.001, ****—*p* < 0.0001. Results are representative of at least two independent experiments.

Given our findings that most HSV-1 membrane proteins are O-glycosylated, and that O-glycans are speculated to play important roles in viral infectivity [37], we wanted to analyze whether O-glycan structures were important for virus production and infectivity. In the past, several studies have used inhibitors of glycosylation that are known to disturb protein trafficking, inhibit growth, and even cause cell death [53–55]. We recently produced an isogenic cell model based on the non-tumorigenic human epidermal keratinocyte cell line, HaCaT [56]. Wild-type HaCaT cells produce mainly core 1 mature ST O-glycans similar to HEL fibroblasts, while HaCaT with *COSMC* knockout, also designated SimpleCells (SC), express glycoproteins with homogenous truncated Tn and STn O-glycans [56]. The isogenic HaCaT cells therefore provide a unique well-defined cellular system to study the effect of truncated O-glycosylation on viral production and infectivity. We infected HaCaT WT and SC in parallel with 10 PFU/cell of HSV-1 Syn17+ produced in HaCaT WT and evaluated viral titers produced in the media. Virus produced in HaCaT SC compared to WT exhibited severely reduced titers as evaluated by plaque assay (on average 10-fold at 12 h and 24-fold at 20 h after infection) (Fig 5A). To evaluate whether this effect was due to production of viral particles we analyzed viral DNA in the media, which showed a substantial reduction in viral DNA (4- to 8-fold at 20 h after infection) detected in the medium from HaCaT SC compared to WT (Fig 5B). These results suggest that truncated O-glycans *per se* pose problems with viral particle formation or early entry events.

## Discussion

Here we provided a strategy for comprehensive characterization of O-glycoproteomes of any virus produced in infected host cells. We demonstrate with the complex model virus HSV-1 that the envelope proteins are heavily O-glycosylated with at least eight membrane proteins being O-glycoproteins. Most of the HSV-1 membrane proteins are also predicted to be N-glycosylated (37 predicted NXS/T sites in total on 11 proteins), although actual N-glycosites in the majority of cases are unknown. In contrast to N-glycosylation, there is a particular need for experimental identification of O-glycosites arising from a lack of simple predictive consensus sequence motifs and the necessity of taking the O-glycosylation capacity of the host cell into account. The key step for sensitive glycoproteomics is enrichment of glycopeptides [57]. For N-glycoproteomics the common N-glycan core structure enables efficient capture of most N-glycopeptides with a mixture of lectins [58, 59], but this is not the case for O-glycans where there is no mixture of lectins available that can encompass all O-glycan structures. Our O-glycoproteomics strategy is therefore versatile for host cells producing core 1 O-glycan structures, but not yet fully applicable to host cells producing more complex O-glycans. However, we show that HSV-1 infection causes an accumulation of truncated O-glycans as well as elongated core 1 structures that can be captured with the available VVA and PNA lectins. The O-glycoproteomic strategy is applicable to any virus produced in infected host cells, which should enable wide application for even highly infectious viruses such as HIV and Ebola.

We chose HSV-1 as a model system because of its complex envelope proteome. Whereas most enveloped viruses in general encode only one or two membrane glycoproteins, the human herpes viruses, including HSV-1, express more than ten glycoproteins located in the viral envelope and various membranes of the infected cells. Human herpes viruses are widely spread pathogens known to establish latency in various cell types enabling recurrent disease by reactivation [60]. HSV-1 is a large DNA virus of high complexity and one of the most prevalent herpes viruses infecting up to 80% of the world’s population [61, 62]. Mature viral particles consist of an icosahedral capsid containing the viral genome, a second less structured protein layer called the tegument, and the surface envelope with at least 12 viral proteins [63]. A number of previous studies have addressed the structure and function of N-linked glycans on HSV-1 glycoproteins [3, 64–66], and there have been attempts to identify and characterize O-glycosylation of proteins as well [45, 67–70]. Although several HSV-1 proteins were previously found to be O-glycosylated, studies of actual O-glycosites are generally missing. We identified multiple O-linked glycosylation sites on HSV-1 proteins important for attachment and entry into target cells (gB, gC, gD, gH, gL) by interactions with host cell receptors such as herpes virus entry mediator (HVEM) [71], nectin-1 [72], 3-O-sulfated heparan sulfate [73], 4-O-sulfated chondroitin sulfate [74], as well as the paired immunoglobulin-like type 2 receptor α (PILRα) [75] and α_V_β_6_/α_V_β_8_ integrins [76]. Other of the identified O-glycans were localized to HSV-1 envelope glycoproteins involved in virus spread or immune modulation (gE, gI, gG) [46, 47, 51]. Of particular interest, we provided confirmation of the glycosylation of the previously identified T53 and T480 sites on gB essential for virus entry in host cells [4, 37, 77]. Based on mutational studies, O-linked glycans at these sites have been specifically implied in gB binding to PILRα [37]. Furthermore, studies in mice indicate that glycosylation at these sites promotes development of keratitis and neuroinvasion [37]. Three of the newly identified sites on gB, T169 and two sites within the peptide stretch 265-YGTT-268, are situated in close proximity to hydrophobic loop regions that are predicted to be involved in fusion with the host membrane, suggesting that the O-glycans could influence the interaction with the host cell [38]. Interestingly, these three sites in gB are found within highly conserved gB regions between the *Herpesviridae* family members of at least seven out of the eight human herpesviruses. Furthermore, a recent study reported that mutational insertion of a fluorescent protein at position 241, which we found to be O-glycosylated, resulted in loss of fusogenic gB function [78]. Another indication that specific O-glycans could be important for interaction between HSV and the host cell was the identification of O-glycosylation sites on glycoprotein D, both within the flexible N-terminus of the molecule that is forming a hairpin upon binding to HVEM (S33) [39, 79], and within the α helix that is part of the interaction surface with the adhesive protein nectin-1 [41] (T255 and Y259/S260). We also identified a cluster of O-glycosites in the HSV-1 glycoprotein C mucin-like domain, which contributes to interaction with glycosaminoglycans on host cells [80, 81]. We did not, however, identify all expected sites in the mucin-like sequences that are notoriously difficult regions to analyze by MS sequencing strategies. Similarly, we did not detect O-glycans in the mucin-like tandem repeat region of gI, which has been shown *in vitro* to accommodate a high level of glycosylation [50].

The glycoproteomic strategy used is based on direct protease digests of virus-infected cells followed by lectin enrichment of O-glycopeptides and ETD-based 'bottom-up' tandem mass spectrometry. With this approach it is possible to address O-glycosylation of viral proteins in a global proteome manner as glycosylated by infected host cells. Since capacity for O-glycosylation varies among cell types, the O-glycoproteome determined in representative infected host cells may guide selection of host cells for recombinant expression of vaccines based on viral membrane proteins. This should be especially important for viruses with high number of O-glycosylation sites such as Ebola virus, Marburg virus, and Crimean-congo hemorrhagic fever virus [25, 82, 83]. It should be noted that the current MS sequencing strategy has some limitations with particularly dense O-glycopeptides with abundant Pro residues. The problem is partly due to difficulties in protease digestion and partly due to insufficient glycopeptide fragmentation in MSn. While this clearly is a limitation, such clustered regions with mucin-like sequence containing high density of PST residues may be reliably predicted to be O-glycosylated in many cell types.

In the intact Golgi apparatus the topology of glycosyltransferases is well organized in different Golgi stacks and TGN in an ordered fashion somewhat reflecting the step-wise biosynthetic pathways of glycosylation [20]. Viral infection is known to induce changes in organization of the Golgi in agreement with the findings of accumulation of truncated O-glycoforms throughout the cytoplasm in the present study [52]. We thus characterized the micro-organization of HSV-1-induced Golgi fragments with respect to different Golgi-compartment resident proteins. Surprisingly, confocal microscopy suggested that the individual Golgi fragments contained the structural components of cis, medial, and trans-Golgi, as demonstrated by highly correlated localization of GM130/giantin and giantin/β4Gal-T1 upon Golgi fragmentation. The existence of cis, medial, and trans-Golgi enzymes within the same fragments would allow sequential O-glycan processing despite Golgi fragmentation, and could explain why we found that infected cells retain the ability to sialylate most of the surface O-linked glycans, regardless of massive amounts of newly synthesized proteins trafficked through a fragmented Golgi apparatus.

A major function of glycosylation of viral envelope glycoproteins appears to be shielding from host immunity [5–7]. The shielding function is well documented for N-glycans [5, 6] but presumably O-glycans serve similar functions [7]. However, the expression of immature truncated O-glycans in the context of virus glycoproteins may have immunostimulatory effects. In contrast to N-glycans with their common large core structure that is highly conserved throughout evolution, the most immature truncated O-glycans are highly immunogenic and may be accommodated together with a short peptide backbone in the binding pocket of an antibody [84]. We and others have previously shown how truncated O-glycopeptides may serve as immunodominant antibody epitopes, which are useful for development of cancer-specific immunotherapeutic intervention and as biomarkers for cancer [85]. In this context, we recently screened a library of short Tn O-glycopeptides covering gG of HSV-2 for the presence of immunodominant O-glycopeptide IgG antibody epitopes in HSV-1 and -2 infected individuals. Interestingly, we did identify one O-glycopeptide epitope to which IgG antibodies were present in HSV-2, but not HSV-1 infected individuals providing a potential diagnostic biomarker [86]. Moreover, the serum IgG antibodies reacted with several glycan structures on the same peptide including truncated (Tn) or elongated (ST) O-glycan, suggesting that these antibodies participate in immunity to viral glycoproteins [86]. The existence of antibodies recognizing O-glycopeptide epitopes suggests that O-glycosylation both with respect to sites and structures should be considered for vaccine design and production. This is especially appealing in relation to targeting patches of O-glycans in mucin domains contained in herpes viruses as well as several human pathogenic virus species, including the deadly Ebola and Marburg viruses. Currently, there are no effective HSV vaccines despite extensive efforts and a better understanding of the O-glycans of the viral glycoproteins may lead to novel approaches for vaccine development.

The widespread nature of O-glycosylation of the HSV-1 envelope proteins prompted us to address the question whether elongated O-glycans are important. We exploited our recently produced isogenic cell model based on the human epidermal keratinocyte cell line HaCaT [56]. Wild-type HaCaT cells express mature core 1 O-glycans while HaCaT SimpleCells (SC) express homogenous truncated Tn and STn O-glycans due to knockout of the private chaperone of the core 1 synthase, C1Gal-T. We used this isogenic cell system to demonstrate that viral propagation and titers in HaCaT SC with truncated O-glycans were severely hampered. Thus, elongated O-glycans are functionally relevant and it is likely that these functions are directed by O-glycans at specific sites in the HSV-1 O-glycoproteome. It should be noted, however, that loss of O-linked glycan elongation has multiple cellular consequences, and further experimentation is required to define the molecular mechanisms behind the observed effect. For this purpose, the HaCaT cell model can now be further explored with glycosyltransferase gene targeted isogenic cell pairs to dissect requirements for particular GalNAc-T repertoire and/or O-glycan structures for HSV-1 viral propagation. It is also conceivable that the dependence on intact O-linked glycosylation for virus generation is not unique to HSV-1, but we anticipate that the described strategy can be used to test the importance of O-glycosylation for other enveloped viruses. A similar genetic deconstruction approach has previously been used with great success for mapping Lassa virus binding to α-dystroglycan and cellular entry [87], and this should greatly advance our understanding of the role of glycosylation in virology.

In summary, we have mapped the O-glycosylation sites on HSV-1 and shown that elongation of O-linked glycosylation is important for HSV-1 biology. Further studies are now possible to decipher the exact mechanism responsible for the observed effects. The glycoproteomics workflow developed should be widely applicable to enveloped viruses with the potential to consider the natural O-glycan coat in the design of antiviral vaccines and drugs.

## Materials and Methods

### Cells and viruses

The wild-type HSV-1 virus Syn17+ [88] was used throughout the study, and the virus titers were determined by plaque titration on Green monkey kidney (GMK, obtained from the Swedish Institute for Infectious Disease Control, Stockholm) cells as previously described [89]. HSV-1 Syn17+ virus was cultivated in HaCaT wild type keratinocytes or GMK cells depending on downstream application and the titers were determined as mentioned above. Diploid human embryonic lung fibroblasts [70] (HEL, obtained from the cell culture collection at the Sahlgrenska University Hospital, department of Clinical Microbiology, Gothenburg) at a low passage level were cultivated in Eagle’s MEM (Gibco, Life Technologies) with 10% FCS (Sigma), 100 IU/mL penicillin, 100 μg/mL streptomycin (Gibco, Life Technologies) and 2 mM L-glutamine. HaCaT wild type [90] and HaCaT *COSMC*-/- [56] keratinocytes were grown in DMEM (Gibco, Life Technologies), supplemented with 10% FCS (HyClone), 100 IU/mL penicillin and 100 μg/mL streptomycin (Gibco, Life Technologies). HaCaT *COSMC*-/- clone D5 harbors a 10 bp deletion at the zink finger nuclease target site of *COSMC* gene, whereas clone E5 harbors a combined 12 bp deletion and a 2 bp insertion. Both genetic alerations result in introduction of STOP codons due to frameshift mutations [56].

### Antibodies and lectins

Monoclonal mouse to Tn (5F4, IgM), mouse to T (3C9, IgM), mouse to STn (3F1, IgG), mouse to GalNAc-T2 (4C4, IgG) mouse to β4Gal-T1 (2F5, IgG) and polyclonal rabbit to gC-1 (KF922, 1:700) antibodies were produced as previously described [24, 91]. Rabbit anti-giantin (1:500) and rat anti-GRP94 (1:50) were purchased from Abcam. FITC-conjugated HPA (*Helix pomatia* agglutinin, 1:2000) was from Invitrogen. FITC-conjugated polyclonal rabbit anti-HSV-1 antibody was purchased from DAKO (1:40). Alexa Fluor 488 F(ab')_2_ fragment of Goat anti-Mouse IgG (H+L) (1:500), Alexa Fluor 546 Goat anti-Mouse IgM (μ chain) (1:500) were from Life Technologies. FITC-conjugated polyclonal Goat anti-Mouse antibody (1:100) and TRITC-conjugated Swine anti-Rabbit antibody (1:200) were from DAKO. Alexa Fluor 647 Goat anti-Mouse IgM (μ chain) was purchased from Life Technologies.

### HSV-1 infection in cell culture

For glycoproteomic analysis, GMK-produced HSV-1 at a multiplicity of infection (MOI) of 3 plaque-forming units (PFU) per cell was added to HEL fibroblasts in roller bottles (34 × 10^6^ cells/bottle). The viral particles were allowed to attach to the cells for 1 h at 37°C and 5% CO_2_ before the inoculum was removed and new growth medium was added. The cells and medium were harvested after most of the cells exhibited cytopathic effects of infection (~20 h). The cells from 3 roller bottles were harvested by scraping with a rubber policeman in ice-cold PBS. The viral particles from the medium were harvested by ultracentrifugation at 100,000 × g for 1 hour at 4°C using 25 × 89 mm ultracentrifuge tubes (Beckman Coulter, Brea, CA) and a Ti70.1-rotor (Beckman Coulter). For glycoprofiling by reductive β-elimination (see S1 Text), confluent HEL fibroblast monolayers (~6 × 10^6^ cells) were infected with MOI of 3 PFU/cell of GMK-produced HSV-1 and harvested at ~23 h post-infection as described above. Medium without serum was used throughout the infection to avoid serum glycan contamination. For immunofluorescence staining, HEL cells were grown either on teflon-coated glass slides or on glass cover slips. Confluent monolayers were infected with GMK-produced HSV-1 at a MOI of 10 PFU/cell as described above. The cells were harvested at either 4 and 8 or 5 and 9 hours post-infection. For infection of keratinocytes, confluent HaCaT wild type or *COSMC*-/- cell monolayers in 6-wells were infected with HaCaT wild type-produced HSV-1 Syn17+ at a MOI of 10 PFU/cell as described above. The growth medium was harvested at 12 and 20 hours after infection.

### LWAC enrichment of Tn and T O-glycopeptides

Infected HEL cell pellet and ultracentrifuged HSV-1 pellet from the growth medium were processed in parallel. The lysates were prepared as previously described [31] with several modifications. Briefly, cell or virus pellet was resuspended in 0.05% RapiGest (Waters) in 50 mM ammonium bicarbonate and lysed using a sonic probe. Cleared cell and virus lysates were reduced and alkylated as described [31] and then treated with 5 U and 1 U, respectively, of PNGase F (Roche) over night at 37°C, followed by digestion with 30 μg/7 μg of trypsin (Roche) for 12 h at 37°C. The PNGase F treatment was then repeated followed by 2 h incubation with 10 μg/3 μg of trypsin. The samples were then treated with concentrated trifluoracetic acid (8 μL/sample, 20 min at 37°C) and cleared by centrifugation (10,000 × g 10 min). The cleared digests were purified on C18 Sep-Pak (Waters) and treated with 100 U of neuraminidase (P0720, New England Biolabs) in 50 mM sodium citrate pH 6.0 at 37°C for 2 h. T and Tn glycopeptides were sequentially enriched using PNA and VVA LWAC as previously described [32] and as described in detail in S1 Text.

### Mass spectrometry

LWAC fractions from total cell lysate digests were screened by preliminary LC-MS for glycopeptide content, and those most enriched in glycopeptides were pooled together and further fractionated by isoelectric focusing as previously described [92]. Mass spectrometry analysis was performed on an EASY-nLC 1000 UHPLC (Thermo Scientific) interfaced via nanoSpray Flex ion source to an LTQ-Orbitrap Velos Pro spectrometer (Thermo Scientific) as previously described [56] with minor changes and as described in detail in S1 Text.

### Data analysis

Data processing was performed using Proteome Discoverer 1.4 software (Thermo Scientific) as previously described with small changes [31]. Due to the high speed of data processing Sequest HT node was used instead of Sequest. All spectra were initially searched with the full cleavage specificity, filtered according to the confidence level (medium, low and unassigned) and further searched with the semi-specific enzymatic cleavage. In all cases the precursor mass tolerance was set to 6 ppm and fragment ion mass tolerance to 50 mmu. Carbamidomethylation on cysteine residues was used as a fixed modification. Methionine oxidation and HexNAc and HexHexNAc attachment to serine, threonine and tyrosine were used as variable modifications for ETD MS2. All HCD MS2 were pre-processed as described [31] and searched under the same conditions mentioned above using only methionine oxidation as variable modification. All spectra were searched against a concatenated forward/reverse human-specific database (UniProt, January 2013, containing 20,232 canonical entries. In addition, another 251 common contaminants and 3187 entries of viruses known to infect humans were included in the search) using a target false discovery rate (FDR) of 1%. FDR was calculated using target decoy PSM validator node, a part of the Proteome Discoverer workflow. The resulting list was filtered to include only peptides with glycosylation as a modification. This resulted in a final glycoprotein list identified by at least one unique glycopeptide. ETD MS2 data were used for unambiguous site assignment. HCD MS2 data were used for unambiguous site assignment only if the number of GalNAc residues was equal to the number of potential sites on the peptide.

### Immunocytochemistry

Teflon-coated glass slides were washed 3 times in PBS followed by 5 min fixation in ice-cold acetone and allowed to air-dry. For cell surface staining, HEL cells grown on cover slips were washed with Hank’s balanced salt solution and fixed with warm 4% paraformaldehyde in PBS for 10 min. Control cells were permeabilized with 0.3% Triton-X100 for 1.5 min. For neuraminidase treatment, teflon-coated slides/cover slips were incubated with 0.1 U/mL *Clostridium perfringens* neuraminidase (Sigma-Aldrich) in 0.05 M sodium acetate pH 5.5 at 37°C for 1 h. Teflon-coated slides/cover slips were incubated with primary antibodies at 4°C over night, washed 3 times with PBS and incubated with secondary antibodies (diluted in 2.5% bovine serum albumin (BSA) in PBS, 0.03% azide) for 45 min in RT. After 3 washes with PBS, the specimens were mounted using ProLong® Gold antifade mounting reagent with 4’,6-diamidino-2-phenylindole (DAPI) (Life Technologies). Immunofluorescent staining was inspected using a Zeiss Axioskop 2 microscope equipped with AxioCam MR3 digital camera. For the co-localization staining using three fluorophores, the teflon-coated glass slides were blocked in 3% BSA in PBS for 30 minutes followed by incubation with primary antibodies and lectins at 4°C over night. Slides were washed 3 times in PBS and once in distilled water followed by incubation with secondary antibodies at 37°C for 45 minutes. Finally the glass slides were washed as described above, air dried and mounted with Prolong Gold Anti-fade reagent containing DAPI (Life Technologies). Triple immunofluorescence staining was analyzed using a Zeiss LSM 510 Meta confocal microscope (Carl Zeiss AG, Oberkochen, Germany) equipped with a Plan-Apochromat 63x objective in oil immersion. Images were edited in Adobe Photoshop CS6.

### Flow cytometry

Confluent HEL cells grown in 6-well plates were infected with HSV-1 at a MOI of 10 or mock infected as described above for indicated time points. The cells were harvested by trypsinization (TrypLE, Life Technologies), washed in 10 mL ice-cold PBS and fixed in 0.1% paraformaldehyde for 24 h at 4°C. After fixation the cells were washed in ice-cold PBS as described above and divided into 100 μL samples with 5 × 10^5^ cells per sample. Half of the samples were permeabilized after fixation by addition of 1x Perm/Wash solution (BD Biosciences) according to the manufacturer’s instructions. After permeabilization, a portion of the HEL cells samples were washed in PBS and treated with 100 μL *Clostridium perfringens* neuraminidase (0.1 U/mL) (Sigma-Aldrich) in 0.05 M sodium acetate pH 5.5 for 40 minutes at 37°C. Thereafter the samples were washed two times in 1x Perm/Wash solution or PBS and incubated with primary antibodies or lectins for 30 min at 4°C. Subsequently the cells were washed as described above and then incubated with secondary antibodies for 30 min at 4°C in the dark followed by washing as described. The cells were analyzed using a Cube8 instrument (Partec Nordic AB) and FlowJo software.

### Plaque assay

Titers of virus produced in HaCaT wild type or *COSMC*-/- keratinocytes were determined on Green monkey kidney (GMK) cells. Cell monolayers were infected with serial dilutions of virus and allowed to attach. After 1 h the inoculum was removed and the cells overlaid with medium containing 1.5% methylcellulose (Sigma-Aldrich), 2.5% FCS, 100 IU/mL penicillin and 100 μg/mL streptomycin (in HBSS (Sigma-Aldrich) + DMEM (Gibco, Life Technologies) at a ratio of 1:1). After 48 h incubation, the overlay medium was removed, the cells fixed with 1% crystal violet (in 70% EtOH:37% formaldehyde:acetic acid 20:2:1), washed three times with water and allowed to dry. The resulting plaques were inspected and counted using a light microscope (Olympus IMT-2).

### Total DNA extraction

The samples were diluted 1:1000 in ice cold PBS and the total DNA content of each diluted fraction was extracted in a MagNa Pure LC robot (Roche Diagnostics, Mannheim, Germany) using a MagNa Pure DNA isolation kit (Roche Diagnostics Scandinavia AB, Stockholm, Sweden), according to the manufacturer’s instructions. The input and the output volumes were adjusted to 200 μL and 100 μL respectively.

### qPCR

For assessing the DNA copy number of HSV-1, a 118-nucleotide segment of the gB-1 region was amplified with primers described in [93]. The PCR reaction volume was set to 50 μL and contained 25 μL TaqMan® 2x PCR Master Mix (Roche Diagnostics, Branchburg, NJ), 15 μL primer/probe mix (forward primer at 0.9 μM, reverse primer at 0.9 μM and probe at 0.2 μM concentrations), and 10 μl of total DNA sample. Amplification of the target sequence was performed using the ABI Prism 7900 system (Applied Biosystems, Foster City, CA). The reaction conditions were set to 2 min at 50°C followed by incubation for 10 min at 95°C and finally 45 PCR cycles of two-step amplification (15 sec at 95°C and 60 sec at 58°C). HSV-1 Forward 5’-GCAGTTTACGTACAACCACATACAGC-3’; HSV-1 Reverse 5’-AGCTTGCGGGCCTCGTT-3’; HSV-1 Probe FAM-5’-CGGCCCAACATATCGTTGACATGGC-3’-TAMRA. The efficiency of each round of PCR was determined using 10-fold dilutions of Topo TA plasmids (Invitrogen AB, Stockholm, Sweden) with insert of respective amplicon created according to the manufacturer’s instructions.

### Statistical analysis

Statistical analysis was performed using GraphPad Prism 6 software.

## Supporting Information

S1 Text Detailed method information. (DOCX)

S1 Fig Gating strategy for the glycoprofiling of HEL cells by flow cytometry. HEL fibroblasts were either mock- or HSV-1-infected (MOI 10) and harvested at indicated time points. Permeabilized or intact cells were double labeled with carbohydrate specific antibodies/lectins and FITC-conjugated HSV-1 antibody (except for HPA-FITC labeled samples) and analyzed by flow cytometry. HPA-FITC—FITC-conjugated *Helix pomatia* lectin (Tn structure (GalNAcα1-O-Ser/Thr)); 3C9 mAb—T structure (Galβ1-3GalNAc1α-O-Ser/Thr); Neu—neuraminidase treatment; hpi—hours post-infection. For the HPA-FITC gating of the permeabilized cells, the majority of the cell population was selected according to side scatter (SSC) and forward scatter (FSC) properties and then the population positive for HPA-FITC stain was selected in the FSC:FL1 plot for visualization as histograms in Fig 1C. HPA-negative cells were de-selected in order to exclude cells not affected by permeabilization, whereas entire populations were selected in the FSC:FL1 plots for visualization in Fig 4E. The gating for the 3C9, 3C9 + Neu, and HSV-1 FITC samples was done by selecting the majority of the population in the SSC:FSC plot and then selecting either the mock- or HSV-1-infected populations in the FSC:FL1 plot, based on the HSV-FITC stain intensity. The resulting populations were depicted in respective histograms in Figs 1C and4E.
(TIF)

S2 Fig Multiple sequence alignment of glycoprotein B between human herpesviruses. ClustalW2 multiple sequence alignment program was used to align amino acid sequences of glycoprotein B ectodomain between the reference strains of members of the *Herpesviridae* family. Output of the multiple sequence alignment is shown. Yellow squares depict identified O-linked glycosylation sites on HSV-1 gB, with red-outlined O-linked glycan icons indicating highly-conserved amino acids between the *Herpesviridae* family members. Ambiguous sites within peptide stretches T109-T123 and T480-S491 are not depicted. Two ambiguous O-glycosylation sites within peptide stretch 265-YGTT-268 were allocated to canonical O-GalNAc acceptor amino acids (T267 and T268). Grey boxes indicate conservation of glycosylated amino acids between the members of *Herpesviridae* family. Black forks indicate protein sequence-predicted N-linked glycosylation sites. HSV-1—human Herpes simplex virus type 1 (strain 17), HSV-2—human Herpes simplex virus type 2 (strain HG52), VZV—Varicella-zoster virus (strain Dumas), HCMV—human cytomegalovirus (strain Merlin), HHV-6—human herpesvirus 6A (strain Uganda-1102), HHV-7—human herpesvirus 7 (strain JI), HHV-8—Kaposi’s sarcoma-associated herpesvirus (isolate GK18), EBV—Epstein-Barr virus (strain AG876).
(TIF)

S3 Fig Co-localization of HSV-1 gC and Tn glycoform. HEL fibroblasts grown on glass slides were infected with HSV-1 Syn17+ at a MOI of 10 and fixed/permeabilized at indicated time points. Mock infected cells were used as control. Cells were double labeled with gC-1 antibody and HPA lectin and analyzed by confocal microscopy in order to investigate O-glycosylation of gC-1 upon HSV-1 infection. Green—HPA (Tn structure (GalNAcα1-O-Ser/Thr)); red—gC-1; blue—DAPI; hpi—hours post infection. Scale bars: 20 μm for lower magnification images and 5 μm for higher magnification images.
(TIF)

S4 Fig Glycoprofiling of HEL fibroblasts by reductive β-elimination. Chemically released glycans from mock- (A, B) or HSV-1 (C, D) infected HEL fibroblasts were analyzed by nano-ESI/MS via direct infusion both at negative (A, C) and positive (B, D) polarities. Peaks representing assigned glycan structures (at least 5% relative abundance) in the spectra are marked. Monoisotopic m/z values, charge state and adduct information are provided. The glycan structures are annotated using the Consortium for Functional Glycomics (CFG) symbol nomenclature (http://www.functionalglycomics.org/static/consortium/Nomenclature.shtml).
(TIF)

S1 Table A list of HSV-1 envelope glycoprotein-derived glycopeptides identified in the MS/MS analysis. The table lists all unique envelope glycoprotein-derived glycopeptides and provides information regarding MS/MS activation type and carbohydrate modifications for individual peptides as well as the cross-correlation score (Xcorr) for each identification. Highest available score is provided. ETD-derived MS/MS spectra were manually inspected for assignment of PTMs. Only correctly assigned O-glycosylation sites are reported as unambiguous in the respective column.
(PDF)

S1 Dataset Indexed reference spectra for infected total cell lysate (PNA). HSV-1 envelope glycoprotein-derived spectra are provided.
(ZIP)

S2 Dataset Indexed reference spectra for infected total cell lysate (PNA flow through VVA). HSV-1 envelope glycoprotein-derived spectra are provided.
(ZIP)

S3 Dataset Indexed reference spectra for HSV-1 particle lysate (PNA). HSV-1 envelope glycoprotein-derived spectra are provided.
(ZIP)

S4 Dataset Indexed reference spectra for HSV-1 particle lysate (PNA flow through VVA). HSV-1 envelope glycoprotein-derived spectra are provided.
(ZIP)
