# Supplementary material for: A Strategy for O-Glycoproteomics of Enveloped Viruses—the O-Glycoproteome of Herpes Simplex Virus Type 1
Source: PLoS Pathog. 2015 Apr 1;11(4):e1004784. doi: 10.1371/journal.ppat.1004784 (PMC4382219; doi:10.1371/journal.ppat.1004784)
Supplement: S1 Text — (DOCX) [file ppat.1004784.s001.docx]

**S1 Text. Detailed method information**

**LWAC enrichment of Tn and T O-glycopeptides**

Infected HEL cell pellet and ultracentrifuged HSV-1 pellet from the growth medium were processed in parallel. Cell pellet was resuspended in 0.05 % RapiGest (Waters) in 50 mM ammonium bicarbonate and lysed using a sonic probe. 0.4 % RapiGest was used for the virus pellet. The lysate was cleared by centrifugation (1,000 × g, 5 min) and the supernatant was heated at 80 °C for 10 min, followed by reduction with 5 mM dithiothreitol (DTT) at 60 °C for 45 min and alkylation with 10 mM iodoacetamide (IAA) at room temperature for 30 min. The cell and virus lysates were then treated with 5 U and 1 U of PNGase F (Roche) over night at 37 °C, respectively, and digested with 30 μg and 7 μg of trypsin (Roche) for 12 h at 37 °C, respectively. The PNGase F treatment was then repeated followed by 2 h incubation with 10 μg and 3 μg of trypsin for the cellular and the virus sample, respectively. The samples were then treated with concentrated trifluoracetic acid (8 μL/sample, 20 min at 37 °C) and cleared by centrifugation (10,000 × g 10 min). The cleared digests were purified on C18 Sep-Pak (Waters) and treated with 100 U of neuraminidase (P0720, New England Biolabs) in 50 mM sodium citrate pH 6.0 at 37 °C for 2 h. Samples were lyophilized, resuspended in 2 mL PNA buffer (10 mM HEPES pH 7.4, 150 mM NaCl, 1 mM CaCl_2_, 0.1 mM MgCl_2_, MnCl_2_ and ZnCl_2_), and injected to a preequilibrated 2.6 m long PNA-agarose (peanut agglutinin-agarose, Vector Labs) column (packed in polytetrafluoroethylene (PFA) tubing 1/16 inch, flow 100 μL min^−1^) for isolation of T O-glycopeptides. The glycopeptides were eluted with 0.5 M D-Gal (1 column volume (CV)), followed by 1 M D-Gal (1 CV) and 1 M D-Gal pH 4 (1 CV) and fractions purified by stage-tips (Thermo Scientific). The flow through fraction was lyophilized and resuspended in VVA buffer (20 mM Tris-HCl pH 7.4, 150 mM NaCl, 1 M urea, 1 mM CaCl_2_, MgCl_2_, MnCl_2_ and ZnCl_2_). The solution was injected to a preequilibrated 2.6 m long VVA-agarose (*Viccia vilosa* agglutinin-agarose, Vector Labs) column (packed in PFA tubing 1/16 inch, flow 100 μL min^−1^) for isolation of Tn O-glycopeptides. The column was first washed with 0.4 M glucose in VVA buffer and then eluted with 0.2 M GalNAc (2 CV) followed by 0.4 M GalNAc (1 CV) and fractions purified by self-made Stage Tips (C18 sorbent from Empore 3M) ([1](#_ENREF_1), [2](#_ENREF_2)).

**Reductive β-elimination**

Cells were lysed followed by reduction and alkylation as described above. Lysates were partially digested with 5 μg of chymotrypsin (Roche) over night at 37 °C. The samples were then treated with concentrated trifluoracetic acid (8 μL/1 mL sample, 20 min at 37 °C) and cleared by centrifugation (10,000 × g 10 min). The cleared digests were purified on C18 Sep-Pak (Waters). Chemical release of O-linked glycans was performed as previously described ([3](#_ENREF_3)). Briefly, reductive β-elimination reactions were performed in 100 μL of 100 mM NaOH and 1 M NaBH_4_ for each sample. Reactions were carried out for 16 h at 50 °C and terminated by addition of 7 μL of glacial acetic acid. Released glycans were separated by passing through C18 Sep-Pak (Waters) columns. Subsequently, Na^+^ ions were removed by Dowex AG 50W X8 (Bio-Rad) cation exchange resin. Glycans were then dried under a stream of nitrogen gas and BH_4_^−^ ions were removed as volatile methyl borate by repeated evaporation (5x) in 500 μL of 1 % acetic acid in methanol. Released glycans were additionally purified by self-made graphitized carbon cartridges ([4](#_ENREF_4)) and analyzed by direct infusion nanoESI-MS (LTQ-Orbitrap Velos Pro, Thermo Scientific) on both positive (soluted in 50 % MeOH 1 % formic acid) and negative (soluted in 50 % MeOH 10 mM ammonium bicarbonate) polarities. Glycan compositional analysis was performed using the SysBioWare platform ([5](#_ENREF_5)).

**Isoelectric focusing**

LWAC fractions from total cell lysate digests were screened by preliminary LC-MS for glycopeptide content, and those most enriched in glycopeptides were pooled together, dried by vacuum centrifugation, reconstituted in IPG rehydration buffer, and submitted to IEF fractionation ([6](#_ENREF_6)). Isoelectric focusing was performed by a 3100 OFFGEL fractionator (Agilent) using pH 3–10 strips (GE Healthcare) 2 fractions were collected and desalted by self-made Stage Tips (C18 sorbent from Empore 3 M) and submitted to LC-MS and HCD/ETD-MS/MS as described below.

**Mass spectrometry**

Mass spectrometry was performed as previously described with minor changes ([7](#_ENREF_7)). EASY-nLC 1000 UHPLC (Thermo Scientific) interfaced via nanoSpray Flex ion source to an LTQ-Orbitrap Velos Pro spectrometer (Thermo Scientific) was used for analysis. The nLC was operated in a single analytical column set up using PicoFrit Emitters (New Objectives, 75 μm inner diameter) packed in-house with Reprosil-Pure-AQ C18 phase (Dr. Maisch, 1.9-μm particle size, 19-21 cm column length). Each sample dissolved in 0.1 % formic acid was injected onto the column and eluted in a gradient from 2 to 20 % B in 95 min, from 20 % to 80 % B in 10 min and 80 % B for 15 min at 200 nL/min (Solvent A, 100 % H_2_O; Solvent B, 100 % acetonitrile; both containing 0.1 % [v/v] formic acid). A precursor MS1 scan (m/z 350–1,700) of intact peptides was acquired in the Orbitrap at a nominal resolution setting of 30,000, followed by Orbitrap HCD-MS2 and ETD-MS2 (m/z of 100–2,000) of the five most abundant multiply charged precursors in the MS1 spectrum; a minimum MS1 signal threshold of 50,000 was used for triggering data-dependent fragmentation events; MS2 spectra were acquired at a resolution of 7,500 for HCD MS2 and 15,000 for ETD MS2. Activation times were 30 and 200 ms for HCD and ETD fragmentation, respectively; isolation width was 4 mass units, and usually 1 microscan was collected for each spectrum. Automatic gain control targets were 1,000,000 ions for Orbitrap MS1 and 100,000 for MS2 scans, and the automatic gain control for fluoranthene ion used for ETD was 300,000. Supplemental activation (20 %) of the charge-reduced species was used in the ETD analysis to improve fragmentation. Dynamic exclusion for 60 s was used to prevent repeated analysis of the same components. Polysiloxane ions at *m/z* 445.12003 were used as a lock mass in all runs.

**References**

1. Steentoft C, Vakhrushev SY, Vester-Christensen MB, Schjoldager KT, Kong Y, Bennett EP, et al. Mining the O-glycoproteome using zinc-finger nuclease-glycoengineered SimpleCell lines. Nature methods. 2011;8(11):977-82.

2. Yang Z, Halim A, Narimatsu Y, Joshi HJ, Steentoft C, Schjoldager KT, et al. The GalNAc-type O-glycoproteome of CHO cells characterized by the SimpleCell strategy. Mol Cell Proteomics. 2014.

3. Jensen PH, Karlsson NG, Kolarich D, Packer NH. Structural analysis of N- and O-glycans released from glycoproteins. Nature protocols. 2012;7(7):1299-310.

4. Packer NH, Lawson MA, Jardine DR, Redmond JW. A general approach to desalting oligosaccharides released from glycoproteins. Glycoconj J. 1998;15(8):737-47.

5. Vakhrushev SY, Dadimov D, Peter-Katalinic J. Software platform for high-throughput glycomics. Analytical chemistry. 2009;81(9):3252-60.

6. Vakhrushev SY, Steentoft C, Vester-Christensen MB, Bennett EP, Clausen H, Levery SB. - Enhanced mass spectrometric mapping of the human GalNAc-type O-glycoproteome with SimpleCells. Mol Cell Proteomics. 2013;12(4):932-44.

7. Radhakrishnan P, Dabelsteen S, Madsen FB, Francavilla C, Kopp KL, Steentoft C, et al. Immature truncated O-glycophenotype of cancer directly induces oncogenic features. Proc Natl Acad Sci U S A. 2014.
