# Supplementary figures and images for: A Strategy for O-Glycoproteomics of Enveloped Viruses—the O-Glycoproteome of Herpes Simplex Virus Type 1
Source: PLoS Pathog. 2015 Apr 1;11(4):e1004784. doi: 10.1371/journal.ppat.1004784 (PMC4382219; doi:10.1371/journal.ppat.1004784)

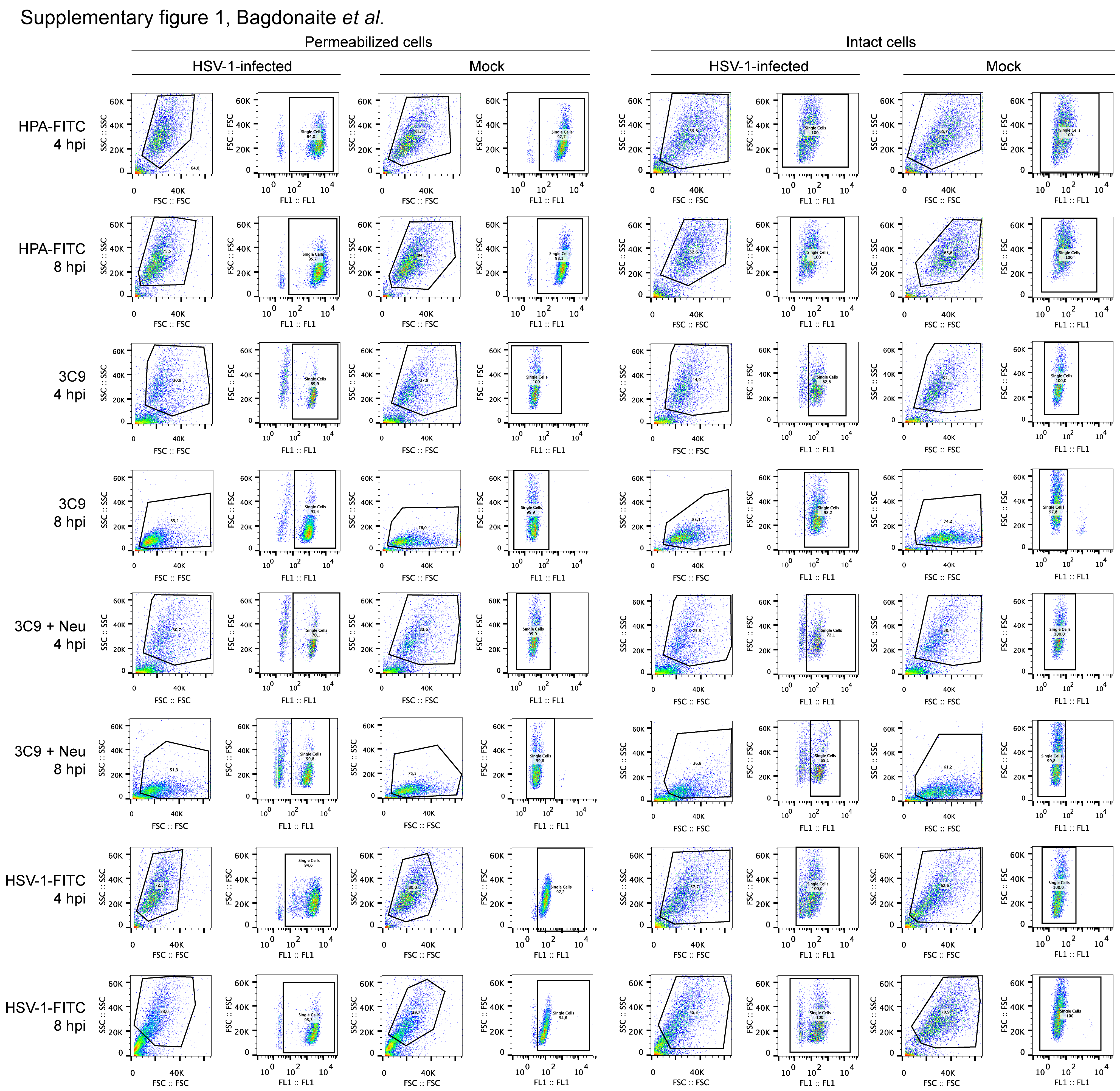

Supplement: S1 Fig — HEL fibroblasts were either mock- or HSV-1-infected (MOI 10) and harvested at indicated time points. Permeabilized or intact cells were double labeled with carbohydrate specific antibodies/lectins and FITC-conjugated HSV-1 antibody (except for HPA-FITC labeled samples) and analyzed by flow cytometry. HPA-FITC—FITC-conjugated Helix pomatia lectin (Tn structure (GalNAcα1-O-Ser/Thr)); 3C9 mAb—T structure (Galβ1-3GalNAc1α-O-Ser/Thr); Neu—neuraminidase treatment; hpi—hours post-infection. For the HPA-FITC gating of the permeabilized cells, the majority of the cell population was selected according to side scatter (SSC) and forward scatter (FSC) properties and then the population positive for HPA-FITC stain was selected in the FSC:FL1 plot for visualization as histograms in Fig 1C. HPA-negative cells were de-selected in order to exclude cells not affected by permeabilization, whereas entire populations were selected in the FSC:FL1 plots for visualization in Fig 4E. The gating for the 3C9, 3C9 + Neu, and HSV-1 FITC samples was done by selecting the majority of the population in the SSC:FSC plot and then selecting either the mock- or HSV-1-infected populations in the FSC:FL1 plot, based on the HSV-FITC stain intensity. The resulting populations were depicted in respective histograms in Figs 1C and4E. (TIF) [file ppat.1004784.s002.tif]

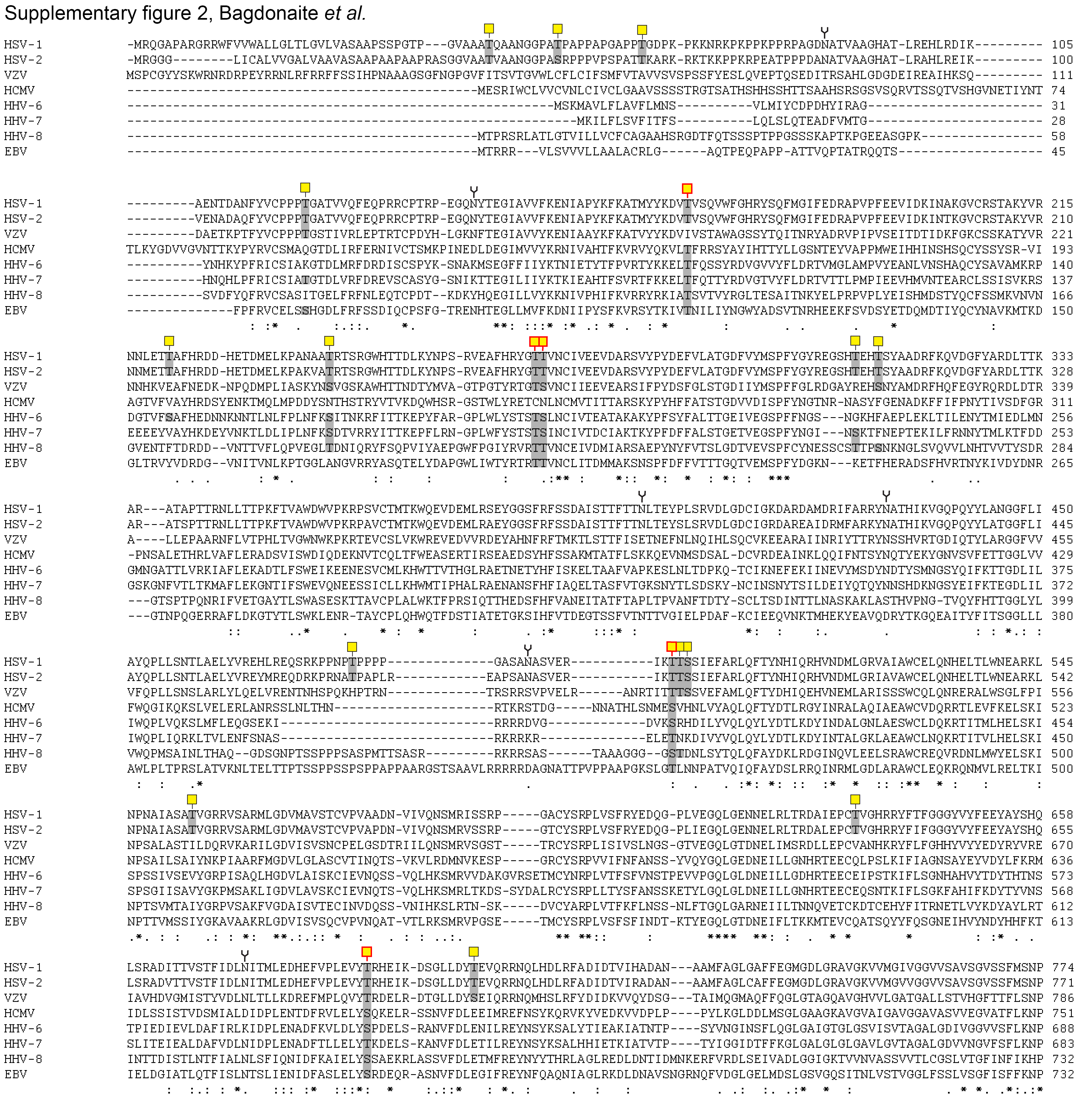

Supplement: S2 Fig — ClustalW2 multiple sequence alignment program was used to align amino acid sequences of glycoprotein B ectodomain between the reference strains of members of the Herpesviridae family. Output of the multiple sequence alignment is shown. Yellow squares depict identified O-linked glycosylation sites on HSV-1 gB, with red-outlined O-linked glycan icons indicating highly-conserved amino acids between the Herpesviridae family members. Ambiguous sites within peptide stretches T109-T123 and T480-S491 are not depicted. Two ambiguous O-glycosylation sites within peptide stretch 265-YGTT-268 were allocated to canonical O-GalNAc acceptor amino acids (T267 and T268). Grey boxes indicate conservation of glycosylated amino acids between the members of Herpesviridae family. Black forks indicate protein sequence-predicted N-linked glycosylation sites. HSV-1—human Herpes simplex virus type 1 (strain 17), HSV-2—human Herpes simplex virus type 2 (strain HG52), VZV—Varicella-zoster virus (strain Dumas), HCMV—human cytomegalovirus (strain Merlin), HHV-6—human herpesvirus 6A (strain Uganda-1102), HHV-7—human herpesvirus 7 (strain JI), HHV-8—Kaposi’s sarcoma-associated herpesvirus (isolate GK18), EBV—Epstein-Barr virus (strain AG876). (TIF) [file ppat.1004784.s003.tif]

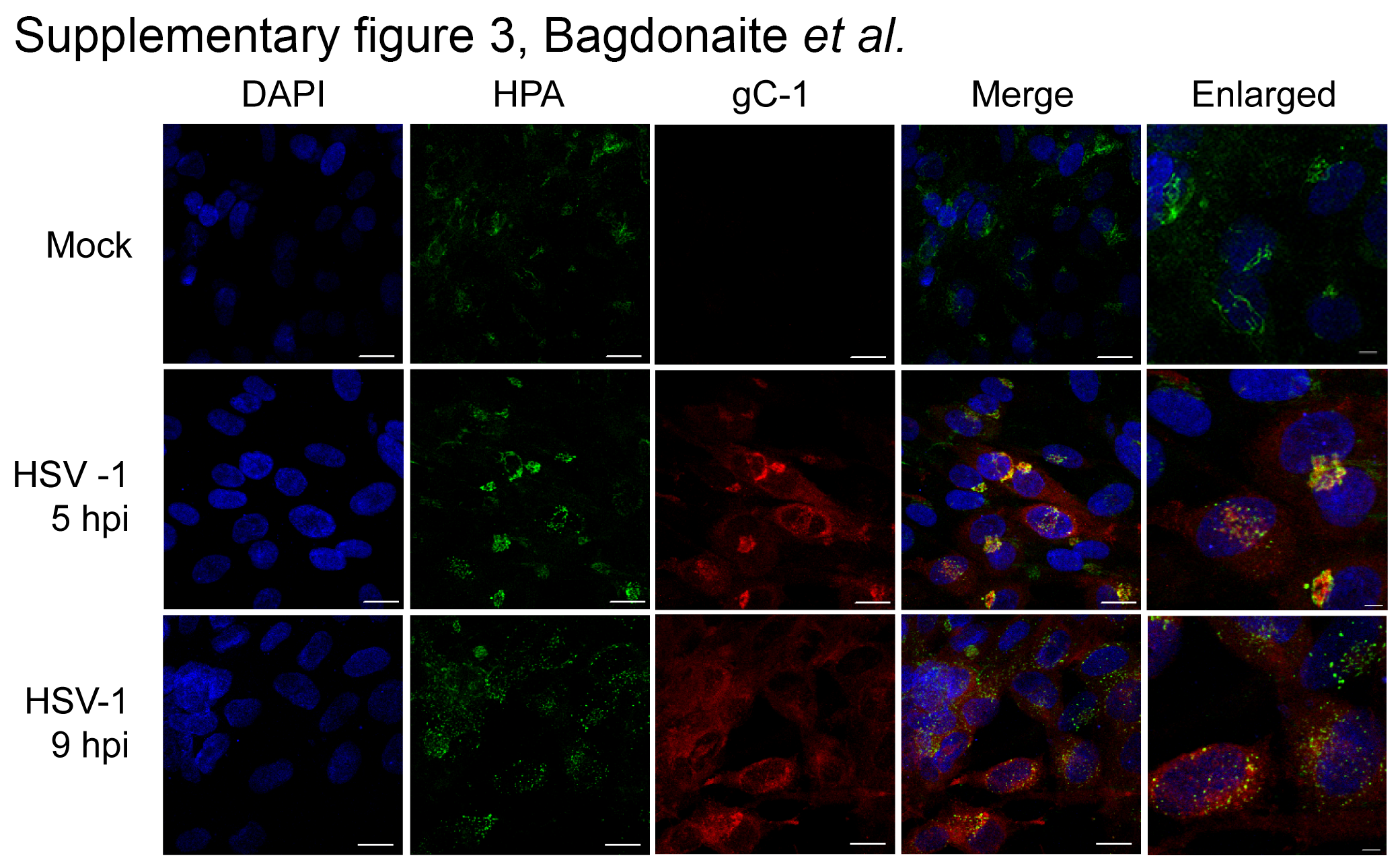

Supplement: S3 Fig — HEL fibroblasts grown on glass slides were infected with HSV-1 Syn17+ at a MOI of 10 and fixed/permeabilized at indicated time points. Mock infected cells were used as control. Cells were double labeled with gC-1 antibody and HPA lectin and analyzed by confocal microscopy in order to investigate O-glycosylation of gC-1 upon HSV-1 infection. Green—HPA (Tn structure (GalNAcα1-O-Ser/Thr)); red—gC-1; blue—DAPI; hpi—hours post infection. Scale bars: 20 μm for lower magnification images and 5 μm for higher magnification images. (TIF) [file ppat.1004784.s004.tif]

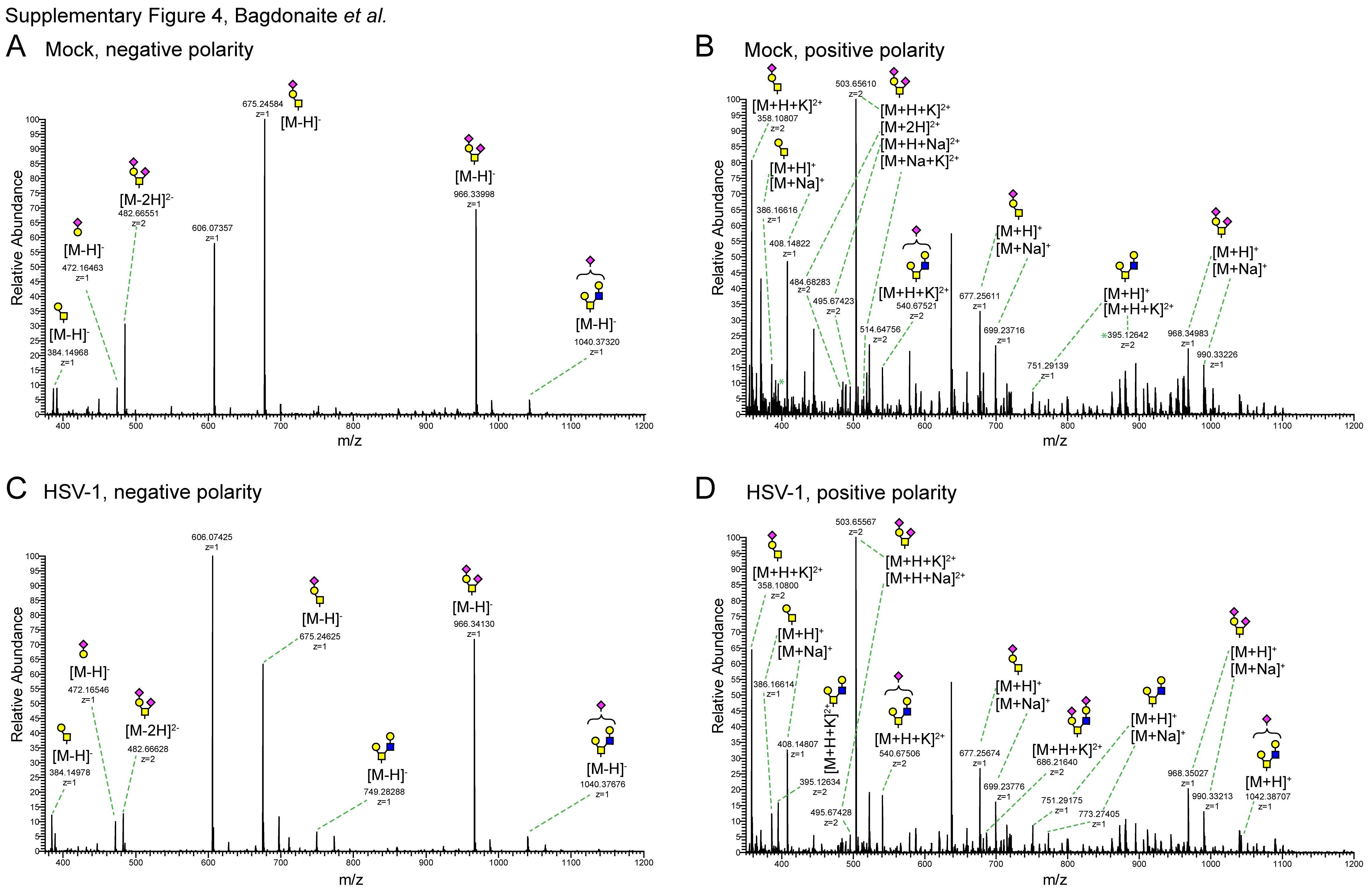

Supplement: S4 Fig — Chemically released glycans from mock- (A, B) or HSV-1 (C, D) infected HEL fibroblasts were analyzed by nano-ESI/MS via direct infusion both at negative (A, C) and positive (B, D) polarities. Peaks representing assigned glycan structures (at least 5% relative abundance) in the spectra are marked. Monoisotopic m/z values, charge state and adduct information are provided. The glycan structures are annotated using the Consortium for Functional Glycomics (CFG) symbol nomenclature (http://www.functionalglycomics.org/static/consortium/Nomenclature.shtml). (TIF) [file ppat.1004784.s005.tif]
