# Supplementary material for: A Strategy for O-Glycoproteomics of Enveloped Viruses—the O-Glycoproteome of Herpes Simplex Virus Type 1
Source: PLoS Pathog. 2015 Apr 1;11(4):e1004784. doi: 10.1371/journal.ppat.1004784 (PMC4382219; doi:10.1371/journal.ppat.1004784)
Supplement: S1 Table — The table lists all unique envelope glycoprotein-derived glycopeptides and provides information regarding MS/MS activation type and carbohydrate modifications for individual peptides as well as the cross-correlation score (Xcorr) for each identification. Highest available score is provided. ETD-derived MS/MS spectra were manually inspected for assignment of PTMs. Only correctly assigned O-glycosylation sites are reported as unambiguous in the respective column. (PDF) [file ppat.1004784.s006.pdf]

S1 Table. A list of HSV-1 envelope glycoprotein-derived glycopeptides identified in the MS/MS analysis.

N-	Sequence[1]	C-	Entry	Gene	Accession	UniProt	Activation type	Modifications	Unambiguous O-linked glycosylation sites	XCorr
31	APSSPGTPGVAAATQAANGGPATPAPPAPGAPPTGDPKPK	70	GB_HHV11	gB	P10211	Envelope glycoprotein B	HCD	2x HexNAc		7.50
31	APSSPGTPGVAAATQAANGGPATPAPPAPGAPPTGDPKPK	70	GB_HHV11	gB	P10211	Envelope glycoprotein B	HCD	3x HexNAc		6.89
33	SSPGTPGVAAATQAANGGPATPAPPAPGAPPTGDPKPK	70	GB_HHV11	gB	P10211	Envelope glycoprotein B	HCD	2x HexNAc		8.20
42	AATQAANGGPATPAPPAPGAPPTGDPKPK	70	GB_HHV11	gB	P10211	Envelope glycoprotein B	HCD	2x HexNAc		5.17
43	ATQAANGGPATPAPPAPGAPPTGDPKPK	70	GB_HHV11	gB	P10211	Envelope glycoprotein B	HCD	2x HexNAc		5.45
43	A**T**QAANGGPA**T**PAPPAPGAPP**T**GDPKPK	70	GB_HHV11	gB	P10211	Envelope glycoprotein B	HCD	3x HexNAc	T44; T53; T64	4.04
44	TQAANGGPATPAPPAPGAPPTGDPKPK	70	GB_HHV11	gB	P10211	Envelope glycoprotein B	HCD	2x HexNAc		5.05
46	AANGGPA**T**PAPPAPGAPP**T**GDPKPK	70	GB_HHV11	gB	P10211	Envelope glycoprotein B	HCD	2x HexNAc	T53; T64	4.93
49	GGPA**T**PAPPAPGAPPTGDPKPK	70	GB_HHV11	gB	P10211	Envelope glycoprotein B	ETD	T5(HexNAc)	T53	3.96
49	GGPATPAPPAPGAPPTGDPKPK	70	GB_HHV11	gB	P10211	Envelope glycoprotein B	HCD	1x HexNAc		4.86
49	GGPA**T**PAPPAPGAPP**T**GDPKPK	70	GB_HHV11	gB	P10211	Envelope glycoprotein B	HCD	2x HexNAc	T53; T64	4.70
49	GGPA**T**PAPPAPGAPP**T**GDPKPK	70	GB_HHV11	gB	P10211	Envelope glycoprotein B	HCD	1x HexNAc; 1x Hex1HexNAc1	T53; T64	3.80
49	GGPA**T**PAPPAPGAPP**T**GDPKPK	70	GB_HHV11	gB	P10211	Envelope glycoprotein B	HCD	2x Hex1HexNAc1	T53; T64	4.86
50	GPA**T**PAPPAPGAPP**T**GDPKPK	70	GB_HHV11	gB	P10211	Envelope glycoprotein B	ETD	T4(HexNAc); T15(HexNAc)	T53; T64	3.33
50	GPA**T**PAPPAPGAPP**T**GDPKPK	70	GB_HHV11	gB	P10211	Envelope glycoprotein B	HCD	2x HexNAc	T53; T64	5.66
50	GPA**T**PAPPAPGAPP**T**GDPKPK	70	GB_HHV11	gB	P10211	Envelope glycoprotein B	HCD	2x Hex1HexNAc1	T53; T64	4.08
51	PA**T**PAPPAPGAPP**T**GDPKPK	70	GB_HHV11	gB	P10211	Envelope glycoprotein B	HCD	2x HexNAc	T53; T64	4.84
53	**T**PAPPAPGAPP**T**GDPKPK	70	GB_HHV11	gB	P10211	Envelope glycoprotein B	ETD	T1(HexNAc); T12(HexNAc)	T53; T64	3.23
53	**T**PAPPAPGAPP**T**GDPKPK	70	GB_HHV11	gB	P10211	Envelope glycoprotein B	HCD	2x HexNAc	T53; T64	4.17
103	DIKAENTDANFYVCPPPTGATVVQFEQPR	131	GB_HHV11	gB	P10211	Envelope glycoprotein B	HCD	1x HexNAc		3.37
103	DIKAENTDANFYVCPPPTGATVVQFEQPR	131	GB_HHV11	gB	P10211	Envelope glycoprotein B	HCD	1x Hex1HexNAc1		6.79
106	AENTDANFYVCPPP**T**GATVVQFEQPR	131	GB_HHV11	gB	P10211	Envelope glycoprotein B	ETD	T15(HexNAc)	T120	4.14
106	AENTDANFYVCPPPTGATVVQFEQPR	131	GB_HHV11	gB	P10211	Envelope glycoprotein B	HCD	1x HexNAc		6.93
106	AENTDANFYVCPPPTGATVVQFEQPRR	132	GB_HHV11	gB	P10211	Envelope glycoprotein B	HCD	1x HexNAc		4.26
108	NTDANFYVCPPPTGATVVQFEQPR	131	GB_HHV11	gB	P10211	Envelope glycoprotein B	HCD	1x HexNAc		6.15
113	FYVCPPPTGATVVQFEQPR	131	GB_HHV11	gB	P10211	Envelope glycoprotein B	HCD	1x HexNAc		5.71
114	YVCPPPTGATVVQFEQPR	131	GB_HHV11	gB	P10211	Envelope glycoprotein B	HCD	1x HexNAc		4.02
115	VCPPP**T**GATVVQFEQPR	131	GB_HHV11	gB	P10211	Envelope glycoprotein B	ETD	T6(HexNAc)	T120	2.40
115	VCPPPTGATVVQFEQPR	131	GB_HHV11	gB	P10211	Envelope glycoprotein B	HCD	1x HexNAc		3.10
106	AENTDANFYVCPPPTGATVVQFEQPR	131	GB_HHV11	gB	P10211	Envelope glycoprotein B	HCD	1x Hex1HexNAc1		7.17
106	AENTDANFYVCPPPTGATVVQFEQPR	131	GB_HHV11	gB	P10211	Envelope glycoprotein B	HCD	2x Hex1HexNAc1		6.11
167	DV**T**VSQVWFGHR	178	GB_HHV11	gB	P10211	Envelope glycoprotein B	ETD	T3(HexNAc)	T169	5.34

[1] Unambiguous O-glycosylation sites within peptide sequences are marked in bold.

167	DVTVSQVWFGHR	178	GB_HHV11	gB	P10211	Envelope glycoprotein B	HCD	1x HexNAc		4.39
216	NNLET**T**AFHR	225	GB_HHV11	gB	P10211	Envelope glycoprotein B	ETD	T6(HexNAc)	T221	2.77
216	NNLETTAFHR	225	GB_HHV11	gB	P10211	Envelope glycoprotein B	HCD	1x HexNAc		2.78
216	NNLETTAFHRDDHETDMELKP ANAATR	242	GB_HHV11	gB	P10211	Envelope glycoprotein B	ETD	T5(HexNAc)		5.47
216	NNLETTAFHRDDHETDMELKP ANAATR	242	GB_HHV11	gB	P10211	Envelope glycoprotein B	HCD	1x HexNAc		5.08
220	TTAFHRDDHETDMELKPANAA **T**R	242	GB_HHV11	gB	P10211	Envelope glycoprotein B	ETD	T22(HexNAc)	T241	6.80
265	YGTTVNCIVEEVDAR	279	GB_HHV11	gB	P10211	Envelope glycoprotein B	HCD	2x HexNAc		4.12
305	EGSH**T**EHTSYAADRFK	320	GB_HHV11	gB	P10211	Envelope glycoprotein B	ETD	T5(HexNAc)	T309	5.56
305	EGSHTEH**T**SYAADRFK	320	GB_HHV11	gB	P10211	Envelope glycoprotein B	ETD	T8(HexNAc)	T312	5.37
305	EGSH**T**EHTSYAADRFK	320	GB_HHV11	gB	P10211	Envelope glycoprotein B	ETD	T5(Hex1HexNAc1)	T309	4.09
305	EGSHTEHTSYAADRFK	320	GB_HHV11	gB	P10211	Envelope glycoprotein B	HCD	1x Hex1HexNAc1		4.74
475	KPPNP**T**PPPP	484	GB_HHV11	gB	P10211	Envelope glycoprotein B	HCD	1x HexNAc	T480	2.71
475	KPPNP**T**PPPPG	485	GB_HHV11	gB	P10211	Envelope glycoprotein B	HCD	1x HexNAc	T480	3.11
475	KPPNP**T**PPPPGA	486	GB_HHV11	gB	P10211	Envelope glycoprotein B	HCD	1x HexNAc	T480	3.42
475	KPPNPTPPPPGAS	487	GB_HHV11	gB	P10211	Envelope glycoprotein B	HCD	1x HexNAc		3.78
475	KPPNPTPPPPGASAN	489	GB_HHV11	gB	P10211	Envelope glycoprotein B	HCD	1x HexNAc		4.43
475	KPPNP**T**PPPPGASANASVER	494	GB_HHV11	gB	P10211	Envelope glycoprotein B	ETD	T6(HexNAc)	T480	4.35
475	KPPNPTPPPPGASANASVER	494	GB_HHV11	gB	P10211	Envelope glycoprotein B	HCD	1x HexNAc		5.16
475	KPPNPTPPPPGASANASVER	494	GB_HHV11	gB	P10211	Envelope glycoprotein B	HCD	2x HexNAc		3.53
475	KPPNP**T**PPPPG	485	GB_HHV11	gB	P10211	Envelope glycoprotein B	HCD	1x Hex1HexNAc1	T480	2.91
475	KPPNP**T**PPPPGA	486	GB_HHV11	gB	P10211	Envelope glycoprotein B	HCD	1x Hex1HexNAc1	T480	3.22
495	IK**TT**SSIEFAR	505	GB_HHV11	gB	P10211	Envelope glycoprotein B	ETD	T3(HexNAc); T4(HexNAc)	T497; T498	3.42
495	IKTTSSIEFAR	505	GB_HHV11	gB	P10211	Envelope glycoprotein B	ETD	T3(Hex1HexNAc1)		2.03
495	IKTT**S**SIEFAR	505	GB_HHV11	gB	P10211	Envelope glycoprotein B	ETD	S5(Hex1HexNAc1)	S499	2.85
495	IK**TT**SSIEFAR	505	GB_HHV11	gB	P10211	Envelope glycoprotein B	ETD	T3(Hex1HexNAc1); T4(Hex1HexNAc1)	T497; T498	3.34
495	IKTTSSIEFAR	505	GB_HHV11	gB	P10211	Envelope glycoprotein B	HCD	1x Hex1HexNAc1		2.76
545	LNPNAIASA**T**VGR	557	GB_HHV11	gB	P10211	Envelope glycoprotein B	ETD	T10(HexNAc)	T554	2.34
545	LNPNAIASATVGR	557	GB_HHV11	gB	P10211	Envelope glycoprotein B	HCD	1x HexNAc		2.35
628	DAIEPC**T**VGHR	638	GB_HHV11	gB	P10211	Envelope glycoprotein B	ETD	T7(HexNAc)	T634	2.82
628	DAIEPCTVGHR	638	GB_HHV11	gB	P10211	Envelope glycoprotein B	HCD	1x HexNAc		3.12
625	LTRDALEPC**T**VGHR	638	GB_HHV11	gB	P10211	Envelope glycoprotein B	ETD	T10(HexNAc)	T634	4.67
625	LTRDALEPCTVGHR	638	GB_HHV11	gB	P10211	Envelope glycoprotein B	HCD	1x HexNAc		4.55
628	DALEPC**T**VGHR	638	GB_HHV11	gB	P10211	Envelope glycoprotein B	ETD	T7(HexNAc)	T634	3.01
628	DALEPC**T**VGHR	638	GB_HHV11	gB	P10211	Envelope glycoprotein B	HCD	1x HexNAc	T634	2.12
628	DALEPC**T**VGHRR	639	GB_HHV11	gB	P10211	Envelope glycoprotein B	ETD	T7(HexNAc)	T634	3.62
678	LEDHEFVPLEVYTR	691	GB_HHV11	gB	P10211	Envelope glycoprotein B	HCD	1x HexNAc		4.83
683	FVPLEVY**T**R	691	GB_HHV11	gB	P10211	Envelope glycoprotein B	ETD	T8(HexNAc)	T690	2.47
692	HEIKDSGLLDY**T**EVQR	707	GB_HHV11	gB	P10211	Envelope glycoprotein B	ETD	T12(HexNAc)	T703	5.33

692	HEIKDSGLLDYTEVQR	707	GB_HHV11	gB	P10211	Envelope glycoprotein B	HCD	1x HexNAc		5.87
692	HEIKDSGLLDY**T**EVQRR	708	GB_HHV11	gB	P10211	Envelope glycoprotein B	ETD	T12(HexNAc)	T703	7.37
692	HEIKDSGLLDYTEVQRR	708	GB_HHV11	gB	P10211	Envelope glycoprotein B	HCD	1x HexNAc		5.24
696	DSGLLDYTEVQR	707	GB_HHV11	gB	P10211	Envelope glycoprotein B	HCD	1x HexNAc		2.50
696	DSGLLDY**T**EVQRR	708	GB_HHV11	gB	P10211	Envelope glycoprotein B	ETD	T8(HexNAc)	T703	1.92
697	SGLLDY**T**EVQR	707	GB_HHV11	gB	P10211	Envelope glycoprotein B	ETD	T7(HexNAc)	T703	2.41
697	SGLLDYTEVQR	707	GB_HHV11	gB	P10211	Envelope glycoprotein B	HCD	1x HexNAc		2.84
76	TTPTEPASPPTTPKP	90	GC_HHV11	GC	P10228	Glycoprotein C	HCD	2x HexNAc		2.51
76	TTPTEPASPPTTPKP**T**	91	GC_HHV11	GC	P10228	Glycoprotein C	ETD	T16(HexNAc)	T91	3.43
76	TTPTEPASPPTTPKPT	91	GC_HHV11	GC	P10228	Glycoprotein C	HCD	1x HexNAc		2.70
76	TTPTEPASPPTTPKPT	91	GC_HHV11	GC	P10228	Glycoprotein C	HCD	2x HexNAc		2.51
76	TTPTEPASPPTTPKPTS	92	GC_HHV11	GC	P10228	Glycoprotein C	HCD	2x HexNAc		2.74
76	TTPTEPASPPT**T**PKPTSTPK	95	GC_HHV11	GC	P10228	Glycoprotein C	ETD	T12(HexNAc)	T87	3.16
76	TTPTEPASPPTTPKP**T**STPK	95	GC_HHV11	GC	P10228	Glycoprotein C	ETD	T16(HexNAc)	T91	4.98
76	**T**TPTEPASPPTTPKPT**S**TPK	95	GC_HHV11	GC	P10228	Glycoprotein C	ETD	T1(HexNAc); S17(HexNAc)	T76; S92	3.44
76	TTPTEPASPPTTPKPT**S**TPK	95	GC_HHV11	GC	P10228	Glycoprotein C	ETD	S8(HexNAc); S17(HexNAc)	S92	2.81
76	TTPTEPASPPT**T**PKP**T**STPK	95	GC_HHV11	GC	P10228	Glycoprotein C	ETD	T12(HexNAc); T16(HexNAc)	T87; T91	3.95
76	TTPTEPASPPTTPKPTS**T**PK	95	GC_HHV11	GC	P10228	Glycoprotein C	ETD	T16(HexNAc); T18(HexNAc)	T93	2.83
76	TTP**T**EPASPPTTPKPTSTPK	95	GC_HHV11	GC	P10228	Glycoprotein C	ETD	T4(HexNAc); T11(HexNAc); T12(HexNAc)	T79	3.77
76	TTPTEPASPP**TT**PKP**T**STPK	95	GC_HHV11	GC	P10228	Glycoprotein C	ETD	T4(HexNAc); T11(HexNAc); T12(HexNAc); T16(HexNAc)	T86; T87; T91	3.19
76	TTPTEPASPP**TT**PKP**T**S**T**PK	95	GC_HHV11	GC	P10228	Glycoprotein C	ETD	T4(HexNAc); T11(HexNAc); T12(HexNAc); T16(HexNAc); T18(HexNAc)	T86; T87; T91; T93	3.82
76	TTPTEPASPPTTPKPTSTPK	95	GC_HHV11	GC	P10228	Glycoprotein C	HCD	1x HexNAc		3.57
76	TTPTEPASPPTTPKPTSTPK	95	GC_HHV11	GC	P10228	Glycoprotein C	HCD	2x HexNAc		3.32
76	TTPTEPASPPTTPKPTSTPK	95	GC_HHV11	GC	P10228	Glycoprotein C	HCD	3x HexNAc		2.21
76	TTPTEPASPPTTPKPTSTPK	95	GC_HHV11	GC	P10228	Glycoprotein C	HCD	4x HexNAc		2.10
78	PTEPASPP**TT**PKP**T**STPK	95	GC_HHV11	GC	P10228	Glycoprotein C	ETD	T9(HexNAc); T10(HexNAc); T14(HexNAc)	T86; T87; T91	3.44
96	SPPTSTPD	103	GC_HHV11	GC	P10228	Glycoprotein C	HCD	2x HexNAc		2.50
96	SPPTSTPDPK	105	GC_HHV11	GC	P10228	Glycoprotein C	HCD	1x HexNAc		2.58
96	SPP**T**S**T**PDPKPK	107	GC_HHV11	GC	P10228	Glycoprotein C	ETD	T4(HexNAc); T6(HexNAc)	T99; T101	2.81
96	SPPTSTPDPKPK	107	GC_HHV11	GC	P10228	Glycoprotein C	HCD	1x HexNAc		4.40
96	SPPTSTPDPKPK	107	GC_HHV11	GC	P10228	Glycoprotein C	HCD	2x HexNAc		3.37
96	SPPTSTPDPKPKN	108	GC_HHV11	GC	P10228	Glycoprotein C	ETD	T4(HexNAc)		3.74
96	SPPTSTPDPKPKN	108	GC_HHV11	GC	P10228	Glycoprotein C	HCD	1x HexNAc		5.45
284	ATCTAAAYYPR	294	GC_HHV11	GC	P10228	Glycoprotein C	HCD	1x HexNAc		3.34
284	ATCTAAAYYPR	294	GC_HHV11	GC	P10228	Glycoprotein C	HCD	1x Hex1HexNAc1		2.90
411	SAV**T**AQESCDHPGLATVR	428	GC_HHV11	GC	P10228	Glycoprotein C	ETD	T4(HexNAc)	T414	5.83
411	SAVTAQESCDHPGLA**T**VR	428	GC_HHV11	GC	P10228	Glycoprotein C	ETD	T16(HexNAc)	T426	4.02
411	SAVTAQESCDHPGLATVR	428	GC_HHV11	GC	P10228	Glycoprotein C	HCD	1x HexNAc		7.29

411	SAVTAQESCDHPGLATVR	428	GC_HHV11	GC	P10228	Glycoprotein C	HCD	2x HexNAc		3.26
26	KYALVDA**S**LK	35	GD_HHV11	gD	Q69091	Envelope glycoprotein D	ETD	S8(HexNAc)	S33	3.34
26	KYALVDA**S**LK	35	GD_HHV11	gD	Q69091	Envelope glycoprotein D	ETD	S8(Hex1HexNAc1)	S33	3.06
26	KYALVDASLK	35	GD_HHV11	gD	Q69091	Envelope glycoprotein D	HCD	1x HexNAc		3.86
26	KYALVDASLK	35	GD_HHV11	gD	Q69091	Envelope glycoprotein D	HCD	1x Hex1HexNAc1		3.26
27	YALVDA**S**LK	35	GD_HHV11	gD	Q69091	Envelope glycoprotein D	ETD	S7(HexNAc)	S33	2.01
27	YALVDASLK	35	GD_HHV11	gD	Q69091	Envelope glycoprotein D	HCD	1x HexNAc		2.76
93	SVLLNAP**S**EAPQIVR	107	GD_HHV11	gD	Q69091	Envelope glycoprotein D	ETD	S8(HexNAc)	S100	3.05
93	SVLLNAP**S**EAPQIVR	107	GD_HHV11	gD	Q69091	Envelope glycoprotein D	ETD	S8(Hex1HexNAc1)	S100	3.06
93	SVLLNAPSEAPQIVR	107	GD_HHV11	gD	Q69091	Envelope glycoprotein D	HCD	1x HexNAc		4.39
93	SVLLNAPSEAPQIVR	107	GD_HHV11	gD	Q69091	Envelope glycoprotein D	HCD	1x Hex1HexNAc1		4.76
93	**S**VLLNAP**S**EAPQIVR	107	GD_HHV11	gD	Q69091	Envelope glycoprotein D	HCD	2x Hex1HexNAc1	S93; S100	4.37
96	LNAP**S**EAPQIVR	107	GD_HHV11	gD	Q69091	Envelope glycoprotein D	ETD	S5(HexNAc)	S100	2.47
96	LNAP**S**EAPQIVR	107	GD_HHV11	gD	Q69091	Envelope glycoprotein D	HCD	1x HexNAc	S100	2.77
98	AP**S**EAPQIVR	107	GD_HHV11	gD	Q69091	Envelope glycoprotein D	HCD	1x HexNAc	S100	2.63
248	FIPENQR**T**VAVYS	260	GD_HHV11	gD	Q69091	Envelope glycoprotein D	ETD	T8(Hex1HexNAc1)	T255	3.46
248	FIPENQR**T**VAVYSLK	262	GD_HHV11	gD	Q69091	Envelope glycoprotein D	ETD	T8(Hex1HexNAc1)	T255	5.15
248	FIPENQRTVAVYSLK	262	GD_HHV11	gD	Q69091	Envelope glycoprotein D	HCD	1x Hex1HexNAc1		3.00
248	FIPENQR**T**VAVYSLK	262	GD_HHV11	gD	Q69091	Envelope glycoprotein D	ETD	T8(HexNAc); S13(Hex1HexNAc1)	T255	2.99
255	**T**VAVYSLK	262	GD_HHV11	gD	Q69091	Envelope glycoprotein D	ETD	T1(HexNAc)	T255	1.97
255	TVAVYSLK	262	GD_HHV11	gD	Q69091	Envelope glycoprotein D	HCD	1x HexNAc		3.03
255	TVAVYSLK	262	GD_HHV11	gD	Q69091	Envelope glycoprotein D	HCD	1x Hex1HexNAc1		2.08
255	TVAVYSLK	262	GD_HHV11	gD	Q69091	Envelope glycoprotein D	HCD	2x HexNAc		2.80
255	TVAVYSLK	262	GD_HHV11	gD	Q69091	Envelope glycoprotein D	HCD	2x Hex1HexNAc1		2.33
29	RVSVGEDVSLLPAPGP**T**GR	47	GE_HHV11	gE	P04488	Envelope glycoprotein E	ETD	T17(HexNAc)	T45	6.16
29	RVSVGEDV**S**LLPAPGP**T**GR	47	GE_HHV11	gE	P04488	Envelope glycoprotein E	ETD	S9(HexNAc); T17(HexNAc)	S37; T45	4.87
29	RV**S**VGEDV**S**LLPAPGP**T**GR	47	GE_HHV11	gE	P04488	Envelope glycoprotein E	ETD	S3(HexNAc); S9(HexNAc); T17(HexNAc)	S31; S37; T45	2.80
29	RVSVGEDV**S**LLPAPGP**T**GR	47	GE_HHV11	gE	P04488	Envelope glycoprotein E	ETD	S9(HexNAc); T17(Hex1HexNAc1)	S37; T45	3.54
29	RVSVGEDVSLLPAPGP**T**GR	47	GE_HHV11	gE	P04488	Envelope glycoprotein E	ETD	T17(Hex1HexNAc1)	T45	4.17
29	RVSVGEDVSLLPAPGPTGR	47	GE_HHV11	gE	P04488	Envelope glycoprotein E	HCD	1x HexNAc		3.27
29	RVSVGEDVSLLPAPGPTGR	47	GE_HHV11	gE	P04488	Envelope glycoprotein E	HCD	1x Hex1HexNAc1		3.19
29	RVSVGEDVSLLPAPGP**T**GRGP**T**QK	52	GE_HHV11	gE	P04488	Envelope glycoprotein E	ETD	T17(HexNAc); T22(HexNAc)	T45; T50	4.52
29	RVSVGEDVSLLPAPGP**T**GRGPTQK	52	GE_HHV11	gE	P04488	Envelope glycoprotein E	ETD	T17(Hex1HexNAc1)	T45	6.21
29	RVSVGEDV**S**LLPAPGP**T**GRGPTQK	52	GE_HHV11	gE	P04488	Envelope glycoprotein E	ETD	S9(Hex1HexNAc1); T17(Hex1HexNAc1)	S37; T45	5.66
29	RVSVGEDVSLLPAPGPTGRGPTQK	52	GE_HHV11	gE	P04488	Envelope glycoprotein E	HCD	1x Hex1HexNAc1		3.70
29	RVSVGEDVSLLPAPGPTGRGPTQK	52	GE_HHV11	gE	P04488	Envelope glycoprotein E	HCD	2x Hex1HexNAc1		4.11
30	VSVGEDVSLLPAPGP**T**GR	47	GE_HHV11	gE	P04488	Envelope glycoprotein E	ETD	T16(HexNAc)	T45	5.43
30	VSVGEDV**S**LLPAPGP**T**GR	47	GE_HHV11	gE	P04488	Envelope glycoprotein E	ETD	S8(HexNAc); T16(HexNAc)	S37; T45	6.56

30	VSVGEDVSLLPAPGP**T**GR	47	GE_HHV11	gE	P04488	Envelope glycoprotein E	ETD	T16(Hex1HexNAc1)	T45	3.86
30	VSVGEDVSLLPAPGPTGR	47	GE_HHV11	gE	P04488	Envelope glycoprotein E	HCD	1x HexNAc		4.21
30	VSVGEDVSLLPAPGPTGR	47	GE_HHV11	gE	P04488	Envelope glycoprotein E	HCD	2x HexNAc		3.96
30	V**S**VGEDV**S**LLPAPGP**T**GR	47	GE_HHV11	gE	P04488	Envelope glycoprotein E	HCD	3x HexNAc	S31; S37; T45	3.44
30	VSVGEDVSLLPAPGPTGR	47	GE_HHV11	gE	P04488	Envelope glycoprotein E	HCD	1x Hex1HexNAc1		3.74
30	VSVGEDVSLLPAPGPTGR	47	GE_HHV11	gE	P04488	Envelope glycoprotein E	HCD	2x Hex1HexNAc1		2.85
30	VSVGEDVSLLPAPGP**T**GRGP**T**Q K	52	GE_HHV11	gE	P04488	Envelope glycoprotein E	ETD	T16(Hex1HexNAc1) ; T21(Hex1HexNAc1)	T45; T50	4.40
30	VSVGEDVSLLPAPGPTGRGPTQ K	52	GE_HHV11	gE	P04488	Envelope glycoprotein E	HCD	3x HexNAc		3.69
30	VSVGEDVSLLPAPGPTGRGPTQ K	52	GE_HHV11	gE	P04488	Envelope glycoprotein E	HCD	2x Hex1HexNAc1		3.51
31	SVGEDVSLLPAPGPTGR	47	GE_HHV11	gE	P04488	Envelope glycoprotein E	HCD	1x HexNAc		3.81
31	SVGEDVSLLPAPGPTGR	47	GE_HHV11	gE	P04488	Envelope glycoprotein E	HCD	1x Hex1HexNAc1		3.00
32	VGEDVSLLPAPGP**T**GR	47	GE_HHV11	gE	P04488	Envelope glycoprotein E	ETD	T14(HexNAc)	T45	3.24
32	VGEDVSLLPAPGPTGR	47	GE_HHV11	gE	P04488	Envelope glycoprotein E	HCD	1x HexNAc		2.96
35	DVSLLPAPGP**T**GR	47	GE_HHV11	gE	P04488	Envelope glycoprotein E	ETD	T11(HexNAc)	T45	2.49
36	VSLLPAPGP**T**GR	47	GE_HHV11	gE	P04488	Envelope glycoprotein E	ETD	T10(HexNAc)	T45	2.27
61	DGCGPLHPSWVSLMPPK	77	GE_HHV11	gE	P04488	Envelope glycoprotein E	HCD	1x Hex1HexNAc1		4.33
91	APVPLAMAYAPPAPSA**T**GGLR	111	GE_HHV11	gE	P04488	Envelope glycoprotein E	ETD	T17(HexNAc)	T107	4.44
91	APVPLAMAYAPPAP**S**A**T**GGLR	111	GE_HHV11	gE	P04488	Envelope glycoprotein E	ETD	S15(HexNAc); T17(HexNAc)	S105; T107	4.49
91	APVPLAMAYAPPAPSA**T**GGLR	111	GE_HHV11	gE	P04488	Envelope glycoprotein E	ETD	T17(Hex1HexNAc1)	T107	3.75
91	APVPLAMAYAPPAPSATGGLR	111	GE_HHV11	gE	P04488	Envelope glycoprotein E	HCD	1x HexNAc		5.96
91	APVPLAMAYAPPAPSATGGLR	111	GE_HHV11	gE	P04488	Envelope glycoprotein E	HCD	2x HexNAc		4.80
91	APVPLAMAYAPPAPSATGGLR	111	GE_HHV11	gE	P04488	Envelope glycoprotein E	HCD	1x HexNAc; 1x Hex1HexNAc1		3.77
91	APVPLAMAYAPPAPSATGGLR	111	GE_HHV11	gE	P04488	Envelope glycoprotein E	HCD	1x Hex1HexNAc1		5.45
91	APVPLAMAYAPPAPSATGGLR	111	GE_HHV11	gE	P04488	Envelope glycoprotein E	HCD	2x Hex1HexNAc1		4.50
91	APVPLAMA**Y**APPAP**S**A**T**GGLR	111	GE_HHV11	gE	P04488	Envelope glycoprotein E	HCD	3x Hex1HexNAc1	Y99; S105; T107	3.23
96	AMAYAPPAPSA**T**GGLR	111	GE_HHV11	gE	P04488	Envelope glycoprotein E	ETD	T12(HexNAc)	T107	2.98
96	AMAYAPPAP**S**A**T**GGLR	111	GE_HHV11	gE	P04488	Envelope glycoprotein E	ETD	S10(HexNAc); T12(HexNAc)	S105; T107	3.11
96	AMAYAPPAP**S**ATGGLR	111	GE_HHV11	gE	P04488	Envelope glycoprotein E	ETD	S10(Hex1HexNAc1)	S105	4.18
96	AMAYAPPAPSA**T**GGLR	111	GE_HHV11	gE	P04488	Envelope glycoprotein E	ETD	T12(Hex1HexNAc1)	T107	3.67
96	AMAYAPPAPSATGGLR	111	GE_HHV11	gE	P04488	Envelope glycoprotein E	HCD	1x HexNAc		3.23
96	AMAYAPPAPSATGGLR	111	GE_HHV11	gE	P04488	Envelope glycoprotein E	HCD	2x HexNAc		2.44
96	AMAYAPPAPSATGGLR	111	GE_HHV11	gE	P04488	Envelope glycoprotein E	HCD	1x Hex1HexNAc1		4.12
98	AYAPPAPSA**T**GGLR	111	GE_HHV11	gE	P04488	Envelope glycoprotein E	ETD	T10(HexNAc)	T107	2.70
98	A**Y**APPAPSA**T**GGLR	111	GE_HHV11	gE	P04488	Envelope glycoprotein E	ETD	Y2(HexNAc); T10(HexNAc)	Y99; T107	2.30
112	**T**DFVWQER	119	GE_HHV11	gE	P04488	Envelope glycoprotein E	HCD	1x HexNAc	T112	2.62
134	ETDSGLYTLSVGDIKDPAR	152	GE_HHV11	gE	P04488	Envelope glycoprotein E	HCD	2x Hex1HexNAc1		4.33
135	TDSGLY**T**LSVGDIKDPAR	152	GE_HHV11	gE	P04488	Envelope glycoprotein E	ETD	T7(HexNAc)	T141	4.25
135	TDSGLYTLSVGDIKDPAR	152	GE_HHV11	gE	P04488	Envelope glycoprotein E	HCD	1x HexNAc		7.05

187	ESLAG**T**PASGTPR	199	GE_HHV11	gE	P04488	Envelope glycoprotein E	ETD	T6(HexNAc)	T192	2.74
187	ESLAGTPASGTPR	199	GE_HHV11	gE	P04488	Envelope glycoprotein E	HCD	1x HexNAc		3.14
188	SLAG**T**PASGTPR	199	GE_HHV11	gE	P04488	Envelope glycoprotein E	ETD	T5(HexNAc)	T192	2.20
200	LPPPPAPPR**S**WPSAPEVSHVR	220	GE_HHV11	gE	P04488	Envelope glycoprotein E	ETD	S10(Hex1HexNAc1)	S209	3.26
209	SWP**S**APEVSHVR	220	GE_HHV11	gE	P04488	Envelope glycoprotein E	ETD	S4(HexNAc)	S212	3.34
209	SWP**S**APEV**S**HVR	220	GE_HHV11	gE	P04488	Envelope glycoprotein E	ETD	S4(HexNAc); S9(HexNAc)	S212; S217	3.37
209	SWP**S**APEVSHVR	220	GE_HHV11	gE	P04488	Envelope glycoprotein E	ETD	S4(Hex1HexNAc1)	S212	3.12
209	SWPSAPEV**S**HVR	220	GE_HHV11	gE	P04488	Envelope glycoprotein E	ETD	S9(Hex1HexNAc1)	S217	3.36
209	SWPSAPEVSHVR	220	GE_HHV11	gE	P04488	Envelope glycoprotein E	HCD	1x HexNAc		3.23
209	SWPSAPEVSHVR	220	GE_HHV11	gE	P04488	Envelope glycoprotein E	HCD	1x Hex1HexNAc1		2.66
209	**S**WPSAPEVSHVRG	221	GE_HHV11	gE	P04488	Envelope glycoprotein E	ETD	S1(HexNAc)	S209	3.08
209	SWPSAPEVSHVRG	221	GE_HHV11	gE	P04488	Envelope glycoprotein E	HCD	1x HexNAc		2.42
210	WPSAPEVSHVR	220	GE_HHV11	gE	P04488	Envelope glycoprotein E	HCD	1x Hex1HexNAc1		4.12
394	GADLAEP**T**HPH	404	GE_HHV11	gE	P04488	Envelope glycoprotein E	HCD	1x HexNAc	T401	4.04
394	GADLAEP**T**HPHVG	406	GE_HHV11	gE	P04488	Envelope glycoprotein E	HCD	1x HexNAc	T401	4.79
394	GADLAEP**T**HPHVG	406	GE_HHV11	gE	P04488	Envelope glycoprotein E	HCD	1x Hex1HexNAc1	T401	4.86
394	GADLAEPTHPHVGAPPHAPPTHGALR	419	GE_HHV11	gE	P04488	Envelope glycoprotein E	HCD	1x Hex1HexNAc1		6.16
101	DTLPQSPGPAFPLAEDVEK	119	GG_HHV11	gG	P06484	Envelope glycoprotein G	HCD	1x Hex1HexNAc1		5.52
101	D**T**LPQ**S**PGPAFPLAEDVEK	119	GG_HHV11	gG	P06484	Envelope glycoprotein G	HCD	2x Hex1HexNAc1	T102; S106	3.22
101	DTLPQSPGPAFPLAEDVEKDKPNRPVVPSPD	131	GG_HHV11	gG	P06484	Envelope glycoprotein G	HCD	1x Hex1HexNAc1		4.81
120	DKPNRPVVP**S**PDPNN	134	GG_HHV11	gG	P06484	Envelope glycoprotein G	HCD	1x Hex1HexNAc1	S129	4.46
132	PNNSPARPE**T**SRPK	145	GG_HHV11	gG	P06484	Envelope glycoprotein G	ETD	T10(Hex1HexNAc1)	T141	5.31
132	PNNSPARPE**TS**RPK	145	GG_HHV11	gG	P06484	Envelope glycoprotein G	ETD	T10(Hex1HexNAc1) ; S11(Hex1HexNAc1)	T141; S142	4.87
146	TPPTIIGPL	154	GG_HHV11	gG	P06484	Envelope glycoprotein G	HCD	1x HexNAc		2.64
146	**T**PP**T**IIGPL	154	GG_HHV11	gG	P06484	Envelope glycoprotein G	HCD	2x HexNAc	T146; T149	2.45
146	TPPTIIGPLATR	157	GG_HHV11	gG	P06484	Envelope glycoprotein G	HCD	1x HexNAc		2.54
146	TPPTIIGPLATR	157	GG_HHV11	gG	P06484	Envelope glycoprotein G	HCD	2x HexNAc		3.82
146	TPPTIIGPLA**T**RPTTR	161	GG_HHV11	gG	P06484	Envelope glycoprotein G	ETD	T4(Hex1HexNAc1); T11(Hex1HexNAc1)	T156	2.93
112	TYLLGRPPNASLPAPTTVEPTAQPPPSVAPLK	143	GH_HHV11	gH	P06477	Envelope glycoprotein H	HCD	3x Hex1HexNAc1		4.83
177	GDNVATASHPSGPR	190	GH_HHV11	gH	P06477	Envelope glycoprotein H	HCD	1x Hex1HexNAc1		3.79
88	VVHVV**T**VTACPR	99	GI_HHV11	gI	P06487	Envelope glycoprotein I	ETD	T6(HexNAc)	T93	3.74
88	VVHVVTV**T**ACPR	99	GI_HHV11	gI	P06487	Envelope glycoprotein I	ETD	T8(HexNAc)	T95	4.07
88	VVHVV**T**V**T**ACPR	99	GI_HHV11	gI	P06487	Envelope glycoprotein I	ETD	T6(HexNAc); T8(HexNAc)	T93; T95	3.06
88	VVHVVTVTACPR	99	GI_HHV11	gI	P06487	Envelope glycoprotein I	HCD	1x HexNAc		3.57
88	VVHVV**T**V**T**ACPR	99	GI_HHV11	gI	P06487	Envelope glycoprotein I	HCD	2x HexNAc	T93; T95	3.14
110	ATDS**T**HSPAYPTLE	123	GI_HHV11	gI	P06487	Envelope glycoprotein I	ETD	T5(HexNAc)	T114	2.39
110	ATDSTHSPAYPTLE	123	GI_HHV11	gI	P06487	Envelope glycoprotein I	HCD	1x HexNAc		3.18
137	A**T**RDYAGVYVLR	148	GI_HHV11	gI	P06487	Envelope glycoprotein I	ETD	T2(Hex1HexNAc1)	T138	3.74

176	TPVLPPDDEAGLQPKPLTTPPPIIATSD	203	GL_HHV11	gL	P10185	Envelope glycoprotein L	HCD	3x HexNAc		3.90
176	TPVLPPDDEAGLQPKPLTTPPPIIATSD	203	GL_HHV11	gL	P10185	Envelope glycoprotein L	HCD	4x HexNAc		3.75
176	TPVLPPDDEAGLQPKPLTTPPPIIATSD	203	GL_HHV11	gL	P10185	Envelope glycoprotein L	HCD	2x Hex1HexNAc1		3.67
185	AGLQPKPL**TT**PPPIIA**TS**DPTPR	207	GL_HHV11	gL	P10185	Envelope glycoprotein L	ETD	T9(HexNAc); T10(HexNAc); T17(HexNAc); S18(HexNAc)	T193; T194; T201; S202	4.46
185	AGLQPKPLTTPPPIIATSDPTPR	207	GL_HHV11	gL	P10185	Envelope glycoprotein L	HCD	2x HexNAc		3.79
185	AGLQPKPLTTPPPIIAT**S**DPTPR	207	GL_HHV11	gL	P10185	Envelope glycoprotein L	ETD	T9(Hex1HexNAc1); S18(Hex1HexNAc1)	S202	3.18
185	AGLQPKPLTTPPPIIATSDPTPR	207	GL_HHV11	gL	P10185	Envelope glycoprotein L	HCD	2x Hex1HexNAc1		4.33
191	PLT**T**PPPIIATSDPTPR	207	GL_HHV11	gL	P10185	Envelope glycoprotein L	ETD	T4(HexNAc)	T194	3.97
191	PLTTPPPIIA**T**SDPTPR	207	GL_HHV11	gL	P10185	Envelope glycoprotein L	ETD	T4(HexNAc); T11(HexNAc)	T201	3.37
191	PL**TT**PPPIIAT**S**DPTPR	207	GL_HHV11	gL	P10185	Envelope glycoprotein L	ETD	T3(HexNAc); T4(HexNAc); S12(HexNAc)	T193; T194; S202	3.08
191	PLTTPPPIIAT**S**DPTPR	207	GL_HHV11	gL	P10185	Envelope glycoprotein L	ETD	T3(Hex1HexNAc1); S12(Hex1HexNAc1)	S202	3.26
191	PLTTPPPIIATSDPTPR	207	GL_HHV11	gL	P10185	Envelope glycoprotein L	HCD	1x HexNAc		6.87
191	PLTTPPPIIATSDPTPR	207	GL_HHV11	gL	P10185	Envelope glycoprotein L	HCD	2x HexNAc		4.37
191	PLTTPPPIIATSDPTPR	207	GL_HHV11	gL	P10185	Envelope glycoprotein L	HCD	3x HexNAc		3.07
193	TTPPPIIAT**S**DPTPR	207	GL_HHV11	gL	P10185	Envelope glycoprotein L	ETD	T1(HexNAc); S10(HexNAc)	S202	3.55
